# Gastric Cancer Epithelial-Mesenchymal Transition-The Role of Micro-RNA

**DOI:** 10.3390/cancers18030462

**Published:** 2026-01-30

**Authors:** Maciej Biskupski, Adam Brachet, Gabriela Hunek, Agnieszka Karabin, Michał Czerski, Wiktoria Bojarska, Robert Karpiński, Grzegorz Teresiński, Alicja Forma, Jacek Baj

**Affiliations:** 1 Chair and Department of Forensic Medicine, Medical University of Lublin, ul. Jaczewskiego 8b, 20-090 Lublin, Polandalicja.forma@umlub.edu.pl (A.F.); 2 Department of Correct, Clinical and Imaging Anatomy, Medical University of Lublin, Jaczewskiego 4, 20-090 Lublin, Poland; d.adam.brachet@umlub.edu.pl (A.B.);; 3 Department of Machine Design and Mechatronics, Faculty of 1 Mechanical Engineering, Lublin University of Technology, Nadbystrzycka 36, 20-618 Lublin, Poland; 4 Institute of Medical Sciences, Faculty of Medicine, The John Paul II Catholic University of Lublin, Konstantynów 1H, 20-708 Lublin, Poland; 5 1st Department of Psychiatry, Psychotherapy and Early Intervention, Medical University of Lublin, Głuska 1, 20-439 Lublin, Poland

**Keywords:** gastric cancer, epithelial-mesenchymal transition, carcinogenesis, micro-RNA, miRNA

## Abstract

Epithelial-mesenchymal transition (EMT) is a key driver of invasion, metastasis, and treatment resistance in gastric cancer, yet its post-transcriptional regulation by microRNAs (miRNAs) is not fully delineated. We performed a structured literature search in PubMed, Web of Science, and Scopus for studies evaluating miRNAs in relation to EMT in gastric cancer and synthesised tumor-intrinsic, microenvironmental, and circulating EMT-related miRNA networks. Downregulated, predominantly tumor-suppressive miRNAs, including miR-34a, miR-200 family, miR-148a, miR-204, miR-30a, miR-101, miR-218, miR-26a, miR-375, miR-506, and others, converge on EMT transcription factors and pathways such as ZEB1/2, Snail, TGF-β/SMAD, Wnt/β-catenin, c-Met, and PI3K/AKT, and their restoration reverses EMT phenotypes in preclinical models. Upregulated oncomiRs, such as miR-21, miR-17-5p, miR-106b-5p, miR-23a, miR-130a-3p, miR-196a-5p, miR-181a, miR-616-3p, miR-301a-3p, miR-150, miR-27a-3p and miR-192/215, target tumor suppressors and reinforce these pathways. Cancer-associated fibroblast, macrophage, neutrophil, and natural killer cell-derived miRNAs, together with systemic indices such as the neutrophil-to-lymphocyte ratio and mediators like FAM3C, add microenvironmental layers of EMT regulation. Several EMT-related miRNAs show consistent associations with invasion, metastasis, peritoneal dissemination, prognosis, and chemoresistance, and many are detectable in circulation. Overall, EMT-related miRNAs orchestrate gastric cancer cell plasticity and tumor-microenvironment crosstalk and represent promising biomarker and therapeutic candidates that warrant validation in prospective, subtype-stratified, and translational studies.

## Simple Summary

Epithelial-mesenchymal transition (EMT) drives invasion, metastasis, immuno-evasion, and therapy resistance in gastric cancer, regulated by a complex network of microRNAs. This review aimed to synthesize current evidence on EMT-related miRNAs and their regulatory roles within tumor cells and the tumor microenvironment, including CAFs, TAMs, TANs, NK cells, and systemic inflammatory mediators. Downregulated tumor-suppressive miRNAs and upregulated oncomiRs form interconnected networks that stabilize EMT, promote tumor plasticity, and accelerate disease progression. Many of these miRNAs are detectable in circulation and correlate with metastasis, prognosis, and therapy response, highlighting their potential as diagnostic, prognostic, and therapeutic biomarkers. Translational studies are needed to validate these candidates, integrate them with molecular subtypes, and guide pathway-directed interventions to improve patient outcomes.

## 1. Introduction

Gastric cancer (GC) is a malignant epithelial neoplasm that arises from the gastric mucosa, constituting a considerable global health challenge [1]. Despite the decreasing incidence rates observed in numerous populations over the past few decades, GC continues to rank as the fifth most diagnosed malignancy. In 2022, it accounted for approximately 968,000 new cases and resulted in 660,000 fatalities worldwide, as documented in the Global Cancer Statistics (GLOBOCAN) database [2]. GC preferentially affects men, with incidence rates about double those observed in women [3]. This gap can be ascribed to a confluence of hormonal, genetic, and lifestyle factors; for example, it has been documented that estrogen confers a protective effect against GC by mitigating inflammation [4]. The determinants of GC’s development can be classified into non-modifiable factors, such as age and genetics, and modifiable risk factors, including smoking, alcohol intake, and obesity [5]. Research reveals that between 1-3% of patients infected with *H. pylori* may develop gastric adenocarcinoma, while less than 0.1% may develop mucosa-associated lymphoid tissue (MALT) lymphoma [6]. This Gram-negative bacterium is categorized as a Class I carcinogen by the World Health Organization [7]. Oncogenesis is indirectly influenced by the activation of an inflammatory response in the stomach mucosa [8]. The development of GC is much worse when this inflammation becomes persistent. Furthermore, it directly influences the process of epithelial-mesenchymal transition (EMT) by causing genetic changes in gastric epithelial cells [9]. Anatomical location, age of onset, and histological or molecular traits can all be used to classify GC. Adenocarcinomas account for about 90% of GC cases [10]. GC can be classified into two categories at the time of diagnosis: conventional GC, which is discovered in patients over 45, and early-onset GC, which is found in people 45 years of age or younger [11]. A molecular categorization of GC has been created by the Cancer Genome Atlas (TCGA) and includes four different subtypes: tumors defined by chromosomal instability (CIN), microsatellite unstable (MSI), genomically stable (GS), and Epstein-Barr virus positive (10% of GCs) [12]. Laurens’ classification includes three subtypes of GC: diffuse GC, which is characterized by poorly cohesive tumor cells that lack glandular differentiation and are more frequently associated with genetic mutations; intestinal GC, which is characterized by glandular structures and frequently associated with environmental risk factors such as chronic *H. pylori* infection; and mixed GC, which combines characteristics from both aforementioned categories [13]. Using this categorization, the results can reveal patterns like the diffuse subtype’s increased resistance to traditional treatments, which may be partially explained by the increased activation of EMT pathways controlled by miRNAs [14]. Furthermore, the diffuse type’s tendency to infiltrate nearby tissues and metastasize earlier than the intestinal type is linked to its poorer prognosis. Because of its anatomical features, the diffuse variety usually has a worse prognosis than the intestinal variant [13]. The conversion of epithelial cells into mesenchymal phenotypes, which increases their invasiveness and resistance to therapeutic interventions, is known as epithelial-mesenchymal transition (EMT), a crucial mechanism in the metastatic process. Recent studies have demonstrated the important involvement of miRNAs as crucial post-transcriptional regulators of EMT in GC, underscoring the complexity of this process’ control. Improved knowledge of EMT and the role of miRNAs may result in new therapeutic targets or diagnostic indicators [15].

Therefore, the purpose of this review is to present a thorough synthesis of the available data on EMT-related miRNAs in GC, combining clinical and experimental data to outline their functions in tumor genesis, progression, metastatic dissemination, and therapeutic resistance. This work aims to map the various EMT-regulating miRNAs in order to highlight their potential as predictive and diagnostic biomarkers, as well as prospective therapeutic targets that could eventually lead to better clinical outcomes for GC patients.

## 2. Epithelial-Mesenchymal Transition

Epithelial-mesenchymal transition (EMT) is a dynamic and reversible cellular program in which epithelial cells progressively lose apico-basal polarity and cell-cell adhesion while acquiring mesenchymal traits such as increased motility, invasiveness, apoptosis resistance, and altered extracellular matrix (ECM) interactions [16,17]. EMT is not a strictly binary switch; tumor cells frequently occupy intermediate (“partial” or hybrid) EMT states that confer high plasticity and support dissemination and therapy tolerance [17,18]. From a physiological perspective, EMT is essential in embryogenesis and tissue remodeling, whereas in cancer it becomes pathologically co-opted to promote invasion, metastatic spread, and relapse [16,17,18]. Conceptually, EMT is often categorized into three types: type 1 (developmental), type 2 (wound healing/fibrosis), and type 3 (tumor-associated EMT) [16,17].

At the molecular level, EMT is characterized by coordinated transcriptional and post-transcriptional reprogramming that suppresses epithelial differentiation while activating mesenchymal and invasive gene expression programs [16,17]. Typical changes include downregulation of epithelial markers (most notably E-cadherin, but also junctional proteins such as claudins) and increased expression of mesenchymal markers including vimentin, N-cadherin, and fibronectin [16,17]. EMT-inducing transcription factors (EMT-TFs) such as Snail/Slug and ZEB1/2 are central executors of this transition, repressing epithelial genes and promoting mesenchymal identity [16,17]. In GC, EMT programs are reinforced by tumor-intrinsic oncogenic signaling as well as microenvironmental cues (inflammation, cytokines, stromal activation), with Wnt/β-catenin and TGF-β pathways representing dominant upstream regulators that converge on EMT-TFs and cytoskeletal remodeling [16,17,19,20,21].

### 2.1. Epithelial-Mesenchymal Transition in Gastric Carcinogenesis

In GC, EMT is widely recognized as a key mechanism underlying local invasion, metastatic dissemination, cancer stemness, and resistance to systemic therapy [16,17,19,20,21]. During EMT, gastric tumor cells undergo profound morphological changes, shifting from an epithelial “cobblestone-like” architecture toward a spindle-shaped phenotype with enhanced motility and invasiveness [16,17]. These phenotypic alterations correspond to actin cytoskeleton reorganization, disruption of tight junctions, weakening of adherens junctions, and loss of epithelial polarity-changes that collectively facilitate tissue infiltration and dissemination [16,17,21].

A pivotal molecular event in GC EMT is functional loss of E-cadherin, resulting in decreased cell-cell adhesion and increased cell detachment potential [16,17]. EMT-TFs (Epithelial-Mesenchymal Transition Transcription Factors) such as ZEB1/2 and Snail/Slug promote this switch by repressing epithelial gene programs and inducing mesenchymal marker expression [16,17]. Importantly, EMT in GC is strongly shaped by non-coding RNA networks, particularly miRNAs that either restrain EMT by suppressing EMT-TFs or enhance EMT by derepressing pro-mesenchymal signaling. For instance, miR-574-3p regulates EMT and cisplatin resistance in gastric carcinoma cells through targeting ZEB1, linking EMT activation directly to chemotherapy failure [22]. In parallel, RUNX3-miR-30a regulatory interactions influence vimentin expression during EMT, highlighting how cytoskeletal remodeling can be controlled through miRNA-mediated circuits [23]. Other miRNAs (e.g., miR-33a) have also been shown to suppress EMT-associated invasion and metastasis through Snail/Slug-dependent mechanisms in GC models [24]. Moreover, dysregulation of miR-200 family circuits-critical for epithelial identity maintenance through repression of ZEB1/2-has been repeatedly implicated in acquisition of invasive properties in GC cells [25]. EMT programs can also be reinforced by growth factor signaling; IGF-I-induced EMT was shown to be restrained by Cbl-b through a ZEB2/miR-200c axis, emphasizing how oncogenic signaling and miRNA regulation converge on EMT control [26].

Extrinsic microenvironmental stimuli further potentiate EMT in gastric carcinogenesis. Chronic inflammation-particularly associated with *H. pylori*-promotes persistent activation of EMT-related cytokine networks and signaling pathways that intensify invasive phenotypes [19,20,21]. Inflammatory mediators (e.g., IL-6, IL-1β, TNF-α) support sustained EMT signaling, while inflammatory remodeling can increase TGF-β activity and other EMT-promoting cues in the tumor niche [19,20,21]. In addition, specific miRNAs can suppress EMT and metastasis by targeting chromatin and transcriptional regulators; miR-2392 was reported to inhibit EMT and metastasis via MAML3 and WHSC1 targeting in GC, underscoring mechanistic diversity in miRNA-EMT control [27].

Functionally, EMT supports metastasis by enabling tumor cells to invade through the ECM and intravasate into lymphatic or blood vessels. EMT-associated ECM remodeling (including induction of proteolytic activity such as MMP upregulation) and increased cellular motility facilitate dissemination and colonization of distant sites [16,17]. Importantly, metastatic establishment often involves partial reversal of EMT through mesenchymal-epithelial transition (MET), highlighting the plasticity and reversibility of these phenotypic programs as a survival strategy across metastatic stages [18].

EMT is also tightly linked to the emergence of cancer stem cell-like phenotypes, which contribute to tumor heterogeneity, metastatic seeding and recurrence [16,17]. Stemness-associated EMT states frequently display high resistance to apoptosis and reduced sensitivity to chemotherapy, reinforcing the view that EMT is a rational therapeutic target in GC [16,17,19]. Collectively, these data support EMT as a central, multi-layered biological process integrating signaling pathway dysregulation, miRNA control and microenvironmental remodeling to drive progression and treatment resistance in GC.

### 2.2. Wnt/β-Catenin Signaling in EMT in GC

The Wnt/β-catenin pathway is one of the best-established EMT-associated signaling axes in GC and a key integrator of transcriptional reprogramming, invasion and stemness [28,29,30]. In canonical Wnt signaling, binding of Wnt ligands to Frizzled/LRP receptors inhibits the β-catenin destruction complex (classically involving APC, AXIN and GSK-3β), enabling β-catenin stabilization and nuclear translocation. In the nucleus, β-catenin cooperates with TCF/LEF transcription factors to activate gene expression programs that promote proliferation, migration and EMT-associated phenotypes [28,30]. In GC, aberrant Wnt/β-catenin activity contributes to EMT by upregulating EMT-TFs (including Snail and ZEB family members), promoting loss of epithelial junctions, and activating mesenchymal and invasion-related transcriptional modules [28,30].

A key feature of Wnt/β-catenin-driven EMT in GC is its close relationship with post-transcriptional regulation by miRNAs. Wnt signaling and miRNAs regulate each other bidirectionally: Wnt pathway activation can alter miRNA expression profiles, while miRNAs can regulate Wnt components and thereby modulate EMT dynamics [28]. In gastric carcinogenesis, miR-192/-215-mediated inhibition of SMG-1 has been shown to promote EMT via activation of Wnt signaling, providing a direct mechanistic example of miRNA-driven Wnt derepression contributing to EMT progression [29]. Such observations are particularly relevant in miRNA-focused analyses, as they illustrate how miRNA perturbations can shift core oncogenic circuitry toward mesenchymal states.

Wnt/β-catenin also functions as a convergence hub linking EMT to tumor aggressiveness and therapeutic resistance [30]. Experimental data support that EMT-like remodeling and Wnt activation can accompany drug-tolerant states; in HER2-positive GC models, trastuzumab-resistant cells exhibited EMT-like features and resistance was promoted by Wnt/β-catenin signaling [31]. Moreover, Wnt pathway modulation influenced trastuzumab sensitivity in patient-derived organoid contexts, supporting a functional role of Wnt-dependent phenotypic states in treatment response [31]. Beyond therapy resistance, Wnt signaling can promote migration and invasion through regulation of EMT marker expression and cytoskeletal dynamics. For example, Kindlin-1 was shown to promote GC cell motility through Wnt/β-catenin pathway activation, enhancing β-catenin nuclear translocation and TCF4 transcriptional activity while modulating EMT markers [32]. Collectively, these findings align with broader evidence that Wnt/β-catenin supports epithelial-mesenchymal plasticity, stemness and treatment failure in GC, and that non-coding RNA networks constitute important regulatory layers within this pathway [30,33].

From a translational perspective, Wnt/β-catenin remains an attractive-yet complex-target due to its fundamental role in normal tissue homeostasis and extensive pathway crosstalk in cancer [30,33]. Therefore, deeper mechanistic understanding of Wnt-EMT integration and its miRNA modulators is essential for identifying tractable intervention nodes aimed at limiting EMT plasticity, metastasis and resistance.

### 2.3. TGF-β Signaling in EMT in GC

Transforming growth factor-β (TGF-β) is widely regarded as an important regulator of EMT, with context-dependent functions that range from growth-inhibitory effects in early tumorigenesis to pro-invasive and pro-metastatic activity in advanced cancers [19,34,35]. TGF-β signaling drives EMT through both canonical Smad-dependent transcriptional programs and multiple Smad-independent signaling branches that reshape cytoskeletal dynamics, cell migration, survival signaling and resistance to apoptosis [34]. In canonical signaling, ligand binding activates TGF-β receptors and induces Smad2/3 phosphorylation, enabling Smad complex formation and transcriptional regulation of EMT effectors, including induction of EMT-TFs such as Snail/Slug and ZEB1/2 [19,34]. However, TGF-β-induced EMT is often stabilized and amplified through non-Smad pathways such as MAPK cascades (ERK/JNK/p38), PI3K/AKT signaling and Rho-family GTPase networks, which reinforce motility and invasive phenotypes [34].

The gastric tumor microenvironment creates a permissive context for TGF-β-driven EMT because inflammation, stromal remodeling and immune dysregulation contribute to persistent EMT-inducing signaling [19,20,21]. In *H. pylori*-associated carcinogenesis, inflammatory remodeling can increase TGF-β activity and suppress epithelial integrity, facilitating EMT initiation in a chronically inflamed niche [19,20,21]. Importantly, cancer-associated fibroblasts (CAFs) can function as major sources of TGF-β and EMT-promoting secreted factors, creating paracrine loops that sustain mesenchymal plasticity [21,35]. Mechanistic evidence in GC supports this concept: a CAF-derived TGF-β/TGILR axis has been shown to mediate crosstalk between CAFs and tumor cells and to drive GC progression, providing direct support for stromal TGF-β signaling as an upstream EMT-promoting mechanism [36].

TGF-β signaling exhibits extensive crosstalk with other EMT-associated pathways, particularly Wnt/β-catenin, which can cooperatively enhance EMT-TF induction and mesenchymal stability [30,34,35]. Therefore, deeper discussion of TGF-β in EMT is clinically relevant: it positions TGF-β-dependent EMT programs as tractable candidates for anti-metastatic strategies and for approaches aimed at limiting phenotypic plasticity and therapy tolerance in GC [17,35,36].

## 3. Micro-RNAs in Carcinogenesis

**Figure 1:**
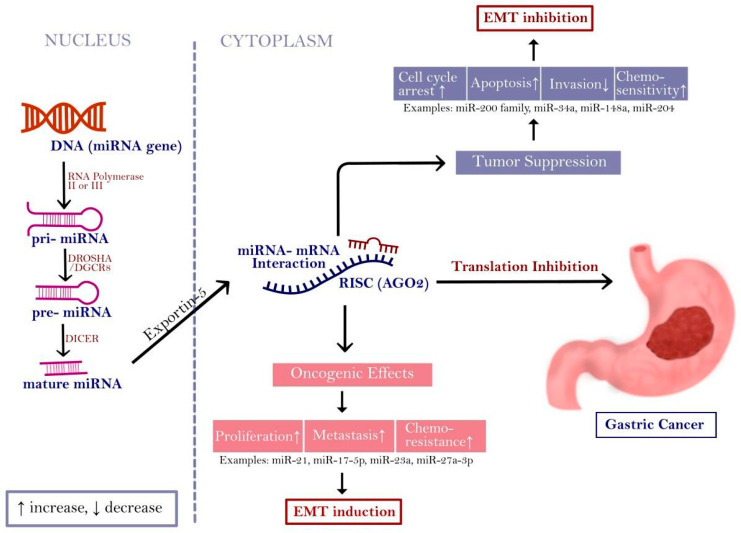
Overview of miRNA biogenesis and functional consequences relevant to EMT in GC.

By attaching to complementary sequences in the 3′-untranslated regions (3′ UTR) of target messenger RNAs (mRNAs), miRNAs-a class of endogenous, single-stranded, short non-coding RNAs that are usually around 22 nucleotides long-control the translation of their target genes [37]. In recent years, thousands of miRNAs have been identified across various organisms, with the human genome now containing over 2500 annotated miRNAs [38,39,40]. MiRNA biogenesis is summarized in Figure 1.

MiRNAs are transcribed as primary transcripts (pri-miRNA) and processed by DROSHA/DGCR8 to precursor miRNAs (pre-miRNA), exported to the cytoplasm and further cleaved by DICER to generate mature miRNAs that act within the RNA-induced silencing complex (RISC) to repress translation of target mRNAs. Depending on context, miRNA dysregulation may promote tumor-suppressive effects (e.g., apoptosis, reduced invasion and increased chemosensitivity) or oncogenic effects (e.g., proliferation, metastasis, chemoresistance) converging on EMT programs in GC. The scheme separates nuclear and cytoplasmic steps and includes Exportin-5-mediated export and RISC (AGO2). Black arrows indicate the direction of the depicted steps/relationships. The dashed vertical line denotes the nuclear-cytoplasmic boundary. ↑ indicates increase and ↓ indicates decrease. Abbreviations: pri-miRNA-primary miRNA; pre-miRNA, precursor miRNA; RISC-RNA-induced silencing complex; EMT-epithelial-mesenchymal transition.

Over half of the miRNA genes are found in genomic regions linked to cancer or in fragile sites [41]. The initial findings on the role of miRNAs in cancer (2002) revealed that two miRNA gene clusters, miR-15a and miR-16a, are situated in the 13q14.3 region, which is often deleted in chronic lymphocytic leukemia (CLL). Further studies (2006) have shown that miR-143 and miR-145 genes, located in the chromosome 5q33 region, are often deleted in lung cancers, resulting in lower expression [42]. Additionally, overexpression of the miR-17-92 cluster has been linked to, e.g., B-cell lymphomas, acute myeloid leukemia (AML), retinoblastoma, or colorectal cancer [43]. Whether changed miRNA expression is a consequence of the pathogenic condition of the malignancy or a contributing cause to its initiation is yet unknown. However, a number of alterations in cancer cells, including chromosomal rearrangements, mutations in miRNA genes, disruptions in proteins involved in their production, and epigenetic modifications, can affect the expression of miRNA [44]. Mutations in non-target mRNA genes may cause miRNA to interact with unwanted mRNA targets, changing their typical regulatory roles. The availability and function of miRNAs can be restricted by epigenetic mechanisms, such as the methylation of miRNA promoter areas or changes in histone charges. MiRNA levels can be altered by mutations or polymorphisms in the miRNA genes themselves, which can either increase or decrease their presence and alter how well they attach to their targets. The correct maturation of miRNAs can also be hampered by disturbances in the biogenesis process of miRNAs, such as variations in DROSHA expression levels. Gene regulation and cellular activity can be impacted by genomic changes that result in elevated or lowered miRNA levels, such as deletions, expansions, or displacements of miRNA loci. Furthermore, miRNA binding efficiency can be altered by mutations or polymorphisms at the target mRNA genes’ miRNA attachment sites, which may reduce the regulatory effect of miRNAs. The intricate ways that miRNA expression and function can be interfered with are demonstrated by these combined mechanisms, which have significant effects on cellular functions [45].

MiRNAs are crucial in the disruption of vital cellular functions in carcinogenesis, including proliferation, invasion and metastasis mechanisms, cell cycle regulation, and resistance to cell death. By targeting important transcription factors, cyclins, cyclin-dependent kinases (Cdks), and their inhibitors, dysregulation of miRNAs greatly contributes to abnormal control of the cell cycle [46]. For instance, the previously discussed miR-17-92 cluster, which normally controls the E2F family of transcription factors (E2F1, E2F2, and E2F3) to guarantee proper cell-cycle transitions, is often overexpressed in a variety of malignancies. Unchecked cell proliferation and tumor growth result from this overexpression’s disruption of the feedback loop regulating E2F activity [47]. Similar to this, miR-221 and miR-222 promote cell-cycle progression by suppressing the Cdk inhibitor p27Kip1, which is frequently seen in glioblastoma and other cancers and correlates with faster tumor growth [48]. Additional Cdk inhibitors, such as p21Cip1 and p16INK4a, are targeted by other miRNAs, including as miR-663 and the miR-302 family, allowing malignant cells to evade crucial cell-cycle checkpoints and promote persistent proliferation [49].

By altering apoptotic pathways, miRNAs not only directly control the cell cycle but also contribute to one of cancer’s defining characteristics: resistance to cell death [50]. By activating anti-apoptotic regulators or suppressing pro-apoptotic proteins via miRNA, tumor cells avoid death. By inhibiting Mdm2, p53-regulated miRNAs, including miR-192, miR-194, and miR-215 stop p53 from being degraded and preserve its tumor-suppressive properties. These miRNAs’ dysregulation promotes survival and carcinogenicity by impairing p53’s reaction to cellular stress [51]. Important anti-apoptotic proteins, including Bcl-2 and Bcl-xL are also modulated by miRNAs. For instance, downregulation of miR-15a and miR-16-1 increases Bcl-2 expression in chronic lymphocytic leukemia, which improves cellular survival [52]. On the other hand, miR-491-5p targets Bcl-xL, to cause apoptosis in ovarian cancer, highlighting the dualistic regulatory potential of miRNAs in cell death pathways [53].

Additionally, by altering important signaling pathways, miRNAs have a significant impact on the growth of tumor cells. For example, downregulation of miR-486 in non-small-cell lung cancer impacts cell migration and growth by targeting elements of the phosphoinositide 3-kinase (PI3K) and insulin-like growth factor (IGF) signaling pathways, demonstrating their ability to reprogram complex signaling networks essential to the development of cancer [54].

Notably, EMT is under tight miRNA control, with certain miRNAs exerting opposing effects: for instance, members of the miR-200 family suppress EMT and help preserve epithelial phenotypes, whereas miRNAs such as miR-155 can promote EMT, enhancing cell motility and invasiveness [55,56]. This bidirectional regulation highlights the complexity of miRNA-driven metastatic programming. The molecular mechanisms through which miRNAs modulate EMT, and the implications of this regulation for cancer progression and metastasis, are discussed in detail in the following section.

## 4. Micro-RNAs in Gastric Cancer Epithelial-Mesenchymal Transition

In the following subsections, we review the role of miRNAs in GC-related EMT at three complementary levels. First, we discuss stromal and immune cell-derived miRNAs and exosome-mediated communication that shape an EMT-promoting tumor microenvironment (including CAF-, TAM-, TAN-, and NK-associated mechanisms). Second, we summarize systemic inflammatory indices and neutrophil-related mediators that have been linked to EMT plasticity and miRNA signalling. Third, we focus on tumor-intrinsic miRNAs that are consistently downregulated or upregulated in GC and act as direct regulators of EMT programs through canonical hubs such as ZEB/E-cadherin, TGF-β/SMAD, and Wnt/β-catenin pathways. Key EMT-associated miRNAs in GC, their principal targets, and functional effects are summarized in Table 1, alongside study design/model, sample type, analytical approach with normalization details, and the main limitations noted in the original reports.

Across these layers, it is helpful to distinguish microenvironmental inputs that act as dominant, sustained EMT enablers versus those that operate as context-dependent amplifiers. In GC, cancer-associated fibroblasts (CAFs) and tumor-associated macrophages (TAMs) frequently appear to impose the most persistent EMT pressure by continuously remodelling the extracellular matrix, secreting EMT-inducing cytokines (e.g., TGF-β, IL-6, IL-8), and exchanging miRNA-rich extracellular vesicles with tumor cells [83,84,85,86,87,88,89,90,91,92,93,94,95,96,97,98,99,100,101,102,103]. In contrast, neutrophil-centred pathways (e.g., IL-17A/NET-driven signalling and neutrophil exosomal miRNAs) and NK-cell dysfunction tend to be more conditional-emerging in inflammatory or immune-evasive contexts and accelerating dissemination once EMT programmes are already primed [104,105,106,107,108,109,110,111,112,113,114,115,116]. Systemic inflammatory indices and mediators such as NLR/PLR and FAM3C can therefore be interpreted as accessible readouts of these tissue-level states, rather than standalone EMT drivers [117,118,119,120,121,122,123,124].

### 4.1. Cancer-Associated Fibroblasts

CAFs are the predominant stromal cell population in many solid tumors and play a critical role in shaping the tumor microenvironment and promoting EMT in GC. CAFs facilitate tumor progression by secreting a wide range of cytokines, growth factors, chemokines, and ECM-remodelling enzymes that collectively support tumor growth, angiogenesis, and therapy resistance. Matrix metalloproteinases (MMPs), TGF-β, vascular endothelial growth factor (VEGF), hepatocyte growth factor (HGF), and IL-22 are important CAF-derived mediators that promote ECM degradation, neovascularization, and EMT induction [83,84,85,86,87,88]. Additionally, CAFs release IL-8, which has been demonstrated to increase chemotherapy resistance in GC by upregulating ABCB1 (P-glycoprotein) and activating the NF-κB pathway, which improves drug efflux [85]. Furthermore, CAFs can promote tumor growth and metastatic spread by inhibiting anti-tumor immune responses [85,89]. Tumor development, progression, and metastasis are all influenced by miRNAs generated from CAF. The ability of GC cells to migrate and invade is increased when miR-214 and miR-139 are downregulated in CAFs. While other miRNAs, such as miR-141 and miR-506, can inhibit migration and angiogenesis in GC, CAF-derived miR-522 has been linked to chemotherapy resistance by decreasing drug sensitivity [83]. Beyond soluble molecules, CAFs mechanically alter the tumor microenvironment by rearranging ECM and adjusting its architecture and rigidity, which promotes tumor invasion. Additionally, they interact extensively with malignant cells. For instance, CAFs release CXCL12, which attaches to tumor cells’ CXCR4 [87,88], encouraging cell motility, assisting EMT, and preventing apoptosis [83]. Tumor cell-released cytokines, such as TNF and interleukins, can activate TGF-β1 signaling, increase CAF motility, and improve CAFs’ capacity to drive GC cell invasion through paracrine processes by stimulating RHBDF2 expression in CAFs [83,89].

Notably, the CAF-dependent miRNA circuits described above should not be viewed in isolation. CAF-derived cytokines and ECM remodelling create a stromal context that cooperates with macrophage polarisation and myeloid recruitment, thereby functionally converging with the TAM- and TAN-linked EMT pathways discussed in Section 4.2 and Section 4.3. This positioning supports a “baseline stromal dominance” model in which CAFs act upstream by establishing an EMT-permissive niche that other immune components subsequently reinforce.

### 4.2. Tumor-Associated Macrophages

Another important part of the GC tumor microenvironment are TAMs, which are essential for angiogenesis, invasion, EMT, and tumor growth [90]. Different polarization states can be adopted by TAMs. While alternatively activated M2 macrophages encourage tumor development, angiogenesis, and immunosuppression, classically activated M1 macrophages exhibit pro-inflammatory, anti-tumor activity [20]. While cytokines like IL-4 and IL-13 produce M2 polarization, triggers like IFN-γ and LPS drive M1 polarization [91]. In GC, cytokines like IL-6 and IL-8 and the activation of pathways like JAK2/STAT3 further encourage macrophage polarization toward the M2 phenotype. These processes promote metastatic spread and increase EMT [92]. PD-1, which interacts with PD-L1 and aids in immune evasion, is often expressed by M2 macrophages and can offset the anti-tumor actions of M1 macrophages [23].

TAMs engage in dynamic interactions with fibroblasts, endothelial cells, and immune cells, among other elements of the tumor microenvironment. They maintain GC progression by interacting with tumor cells, T lymphocytes, vascular endothelial cells, CAFs, and mesenchymal stem cells through the release of extracellular vesicles, cytokines, chemokines, and growth factors [90]. TAM-derived cytokines that stimulate angiogenesis, lymphangiogenesis, and tumor development include VEGF, IL-10, CCL5, and CXCL8 (IL-8). A worse prognosis and increased expression of VEGF and VEGF-C are linked to increased macrophage infiltration [93]. By encouraging EMT and cellular change, macrophage migration inhibitory factor (MIF) connects gastric carcinogenesis to persistent inflammation. TAMs also trigger several molecular pathways, such as FOXQ1, β-catenin, and hypoxia-inducible factor-1α (HIF-1α), that promote EMT and metastasis. GC cell invasiveness is increased when TAMs activate β-catenin signaling, indicating that this pathway may be therapeutically beneficial [94]. EMT induction in GC has been linked to increased TAM infiltration and HIF-1α activation [95]. Furthermore, FOXQ1, which improves tumor cell migration and invasiveness and encourages EMT, is upregulated by TAMs [94]. M2 TAMs promote immunosuppression in the tumor microenvironment from an immunological standpoint. By secreting TGF-β, they inhibit natural killer (NK) cell function and reduce NK-cell numbers within tumors, weakening anti-tumor immunity [20]. M2 TAMs also indirectly suppress cytotoxic T-cell activity through direct cell-cell interactions and by secreting chemokines such as CCL5, CCL20, and CCL22, which modulate T-cell recruitment and function [91]. The combined suppression of NK and T cells allows tumors to evade immune surveillance and promotes progression.

TAMs communicate bidirectionally with GC cells through exosomal miRNAs, thereby shaping an EMT-permissive and therapy-resistant microenvironment. GC cells can “educate” infiltrating macrophages by exporting specific miRNAs in exosomes. For example, GC-cell-derived exosomal miR-15b-5p promotes M2 polarization of TAMs via suppression of WIF1 and activation of WNT5A-a non-canonical Wnt ligand linked to EMT and invasive behaviour in multiple tumors [96]. Similarly, exosomal miR-541-5p released by GC cells induces M2 polarization through the DUSP3/JAK2/STAT3 pathway, and enhances the proliferation and migration of GC cells, further reinforcing EMT-associated malignant traits [97].

Conversely, M2-polarized TAMs also transfer oncogenic miRNAs back to GC cells. Exosomes derived from M2 macrophages are enriched in miR-487a, which is efficiently taken up by GC cells and downregulates the RNA-binding protein TIA1, thereby promoting tumor cell proliferation and tumorigenesis in vitro and in vivo [98]. Another key example is exosomal miR-588 from M2 macrophages, which confers cisplatin resistance to GC cells by targeting CYLD and reducing apoptosis [99]. TAM-derived exosomal miR-21 similarly decreases cisplatin sensitivity of GC cells, at least in part via modulation of the PTEN/PI3K/AKT pathway, linking macrophage-cancer cell signalling to both survival and EMT programmes [100].

In addition to exosome-mediated transfer, TAM-induced inflammatory cytokines can reshape EMT-related miRNA expression within cancer cells. CD204-positive M2-like TAMs increase GC cell migration and EMT by upregulating miR-210 via TNF-α/NF-κB/HIF-1α signalling; miR-210 directly suppresses netrin-4 (NTN4), facilitating motility and mesenchymal transition of GC cells [101]. At the same time, hypoxia in the GC microenvironment downregulates miR-30c in tumor-infiltrating macrophages, leading to reduced mTOR activity, impaired glycolysis, and a lower proportion of pro-inflammatory M1 cells, thereby favouring an immunosuppressive, EMT-supporting TAM phenotype [102]. Collectively, these data indicate that TAM-related miRNAs mediate a reciprocal crosstalk that promotes EMT, invasion, and chemoresistance in GC, positioning exosomal miRNA circuits as attractive candidates for therapeutic targeting [103].

TAM-driven EMT reinforcement is mechanistically compatible with CAF-mediated matrix remodelling and cytokine release (Section 4.1), together forming self-reinforcing stromal loops that stabilise EMT transcriptional programmes in tumor cells [83,84,85,86,87,88,89,90,91,92,93,94,95,96,97,98,99,100,101,102,103]. Taken together, these data suggest that TAM activity is often a dominant sustaining force for EMT once stromal signalling is established, rather than a strictly isolated driver.

### 4.3. Tumor-Associated Neutrophils

Tumor-associated neutrophils (TANs) accumulate at the invasive margin of gastric tumors and correlate with advanced TNM stage, lymphovascular and perineural invasion, and poor disease-free and disease-specific survival [104,105]. Functionally, TANs adopt a predominantly pro-tumorigenic N2 phenotype that secretes cytokines, chemokines, and proteases, thereby promoting angiogenesis, immunosuppression, and metastatic dissemination [105]. In GC, one of the best-characterized mechanisms linking TANs to EMT is the production of IL-17A. Li et al. demonstrated that CD66b^+^ TANs are a major source of IL-17A in gastric tumors and that TAN-conditioned media enhance migration, invasion, and EMT of GC cells; this effect is mediated by activation of the JAK2/STAT3 pathway and is accompanied by decreased E-cadherin and increased vimentin and ZEB1 expression [104]. Neutralization of IL-17A or pharmacologic inhibition of JAK2/STAT3 with AG490 reversed these EMT-associated changes and reduced TAN-induced motility of GC cells [104]. These data support a model in which TAN-derived IL-17A acts upstream of canonical EMT signalling nodes that are themselves regulated by multiple EMT-related miRNAs within tumor cells.

Beyond soluble cytokines, TANs promote EMT and metastasis through the formation of neutrophil extracellular traps (NETs). NETs are web-like DNA structures decorated with histones, neutrophil elastase, and matrix metalloproteinases that can physically entrap circulating tumor cells and deliver pro-invasive cues [105,106]. NETs were shown to induce a more aggressive mesenchymal phenotype in GC cells, with reduced epithelial markers, increased vimentin expression, and enhanced migratory and invasive capacity; pharmacological or enzymatic disruption of NETs attenuated EMT and reduced distant metastases [106]. Clinical data further indicate that postoperative infectious complications are associated with increased NET formation and higher rates of recurrence in GC patients, suggesting that TAN-NET activity can amplify EMT and metastatic risk in vulnerable perioperative windows [107]. Although the direct miRNA control of NET formation has not yet been fully defined in GC, experimental work in other models indicates that NET-associated pathways are modulated by non-coding RNAs, implying that NET-driven EMT may intersect with miRNA-regulated transcriptional networks controlling cytoskeletal reorganisation and cell-matrix interactions [108].

A more direct connection between neutrophils, miRNAs, and EMT in GC arises from exosome-mediated communication. Zhang et al. demonstrated that N2-polarised TANs secrete exosomes enriched in miR-4745-5p and miR-3911, which are efficiently transferred to GC cells [109]. These neutrophil-derived exosomal miRNAs converge on SLIT2, a secreted axon-guidance molecule with tumor-suppressive properties; downregulation of SLIT2 by miR-4745-5p/miR-3911 enhances GC cell migration, invasion, EMT marker expression, and metastatic colonisation in vivo [109]. Restoration of SLIT2 expression reverses these phenotypes, supporting a causal role for the TAN exosomal miRNA-SLIT2 axis in EMT-driven metastasis [109]. Complementing these mechanistic data, a separate clinical study showed that circulating CD66b^+^ neutrophil-derived exosomes and their miRNA cargo, particularly miR-223-3p, have high diagnostic accuracy for distinguishing GC patients from healthy and benign-disease controls [110]. Although miR-223-3p was primarily evaluated as a biomarker, it has established roles in myeloid differentiation and inflammatory signalling [110], suggesting that neutrophil-derived exosomes miRNA signatures may simultaneously reflect and contribute to EMT-permissive inflammatory circuits in the tumor microenvironment.

Taken together, these findings indicate that TANs foster GC EMT through at least three interconnected mechanisms: IL-17A-JAK2/STAT3 signalling, NET formation, and exosomal delivery of pro-metastatic miRNAs. Compared with the continuous, structurally anchored EMT pressure exerted by CAFs and TAMs (Section 4.1 and Section 4.2), TAN-related EMT regulation frequently appears more inducible and context-dependent, driven by episodic IL-17A signalling, NET formation, and exosomal miRNA transfer during heightened inflammation [104,105,106,107,108,109,110]. This framework aligns with clinical observations linking systemic inflammatory metrics (Section 4.5) to aggressive clinicopathological features, consistent with a myeloid-dominant environment that amplifies EMT in susceptible tumors [117,118,119]. TANs may therefore act as accelerators of invasion and metastatic efficiency rather than uniform EMT initiators across all GC contexts.

### 4.4. Natural Killer Cells

Natural killer (NK) cells are key effectors of innate antitumor immunity, capable of directly lysing malignant cells and shaping adaptive responses through cytokine secretion. In GC, higher NK-cell infiltration generally correlates with more favourable outcomes, whereas advanced, immunosuppressed tumors often show reduced NK activity and an EMT-like phenotype, characterised by loss of epithelial markers, increased motility, and altered expression of NK-activating ligands [111,112]. Recent data indicate that this functional impairment of NK cells is tightly linked to non-coding RNA networks, including tumor- and NK-derived miRNAs, which modulate immune checkpoint expression, cytotoxic granule release, and survival of NK cells in the GC microenvironment [112,113,114].

A central mechanism connecting miRNAs, NK dysfunction, and EMT in GC is exosome-mediated transfer of tumor-derived miR-552-5p. Tang et al. showed that GC cell-derived exosomes enriched in miR-552-5p are internalised by NK cells, where miR-552-5p downregulates PD-1/PD-L1 axis components and NK-activating receptors (NKG2D, NKp46), leading to reduced production of interferon-γ, granzyme B, and perforin [111]. Building on this, Qin et al. demonstrated that exosomal miR-552-5p not only dampens NK-cell cytotoxicity but also promotes EMT: NK cells exposed to Exo-miR-552-5p lose effector function, and co-cultured tumor cells show decreased E-cadherin and increased N-cadherin and vimentin expression, along with enhanced migration, invasion, and lung metastasis in mouse models [113]. Blockade of PD-L1 restored NK-cell activity and partially reversed EMT marker changes in tumor tissues, supporting a model in which the exosomal miR-552-5p-PD-1/PD-L1 axis links immune escape to EMT-driven progression [111,113].

Non-coding RNAs intrinsically expressed in NK cells also modulate their antitumor function via miRNA-dependent mechanisms. In peripheral NK cells from patients with GC, the long non-coding RNA GAS5 is downregulated, while its target miR-18a is upregulated [115]. Forced expression of GAS5 in NK cells suppresses miR-18a, increases expression of granzyme B and perforin, enhances NK-mediated cytotoxicity against GC cells, and elevates IFN-γ secretion [115]. Although this study did not directly quantify EMT markers, the restoration of NK effector function is mechanistically linked to reduced tumor cell survival and invasion, processes that are tightly coupled to EMT programmes in GC. GAS5/miR-18a therefore, represents a prototypical example of an lncRNA-miRNA axis that can be therapeutically manipulated to enhance NK-cell activity and indirectly constrain EMT [112,115].

GC cells can also induce qualitative loss of NK cells through ncRNA-regulated ferroptosis. Li et al. reported that tumor-derived exosomal circPDSS1 is transferred to NK cells, where it acts as a competing endogenous RNA for miR-1278, relieving repression of glutamic-oxaloacetic transaminase 1 (GOT1) and promoting ferroptotic cell death in NK cells [116]. Depletion of circPDSS1 or restoration of miR-1278 in NK cells reduced GOT1 expression, attenuated ferroptosis, and improved NK-cell viability and cytotoxicity against GC cells in vitro and in xenograft models [116]. Functionally, loss of NK surveillance in this context facilitates outgrowth of more aggressive, EMT-like tumor clones; in line with this, circPDSS1 overexpression in tumor-derived exosomes is associated with increased expression of mesenchymal markers and higher metastatic burden in vivo [116]. Exosomal circPDSS1-miR-1278-GOT1 thus defines another ncRNA circuit by which GC cells disable NK-mediated control and create permissive conditions for EMT and dissemination.

**Figure 2:**
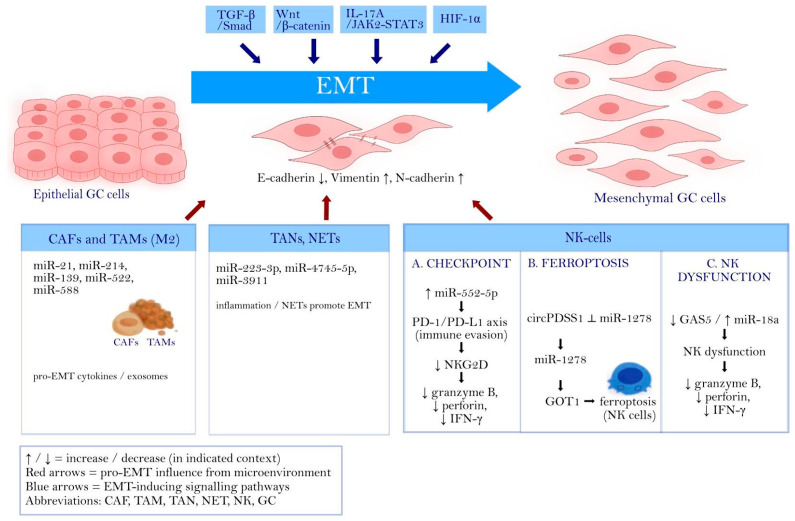
miRNA-regulated EMT in GC across tumor and microenvironmental compartments.

Collectively, these studies indicate that miRNA-centred networks play a dual role in regulating NK cells and EMT in GC: (1) GC-derived exosomal miRNAs (e.g., miR-552-5p, circPDSS1/miR-1278) impair NK-cell cytotoxicity via immune checkpoints and ferroptosis, thereby allowing EMT-driven tumor cells to expand; and (2) NK-intrinsic ncRNA axes (e.g., GAS5/miR-18a) can be harnessed to restore NK function and indirectly suppress EMT. Targeting these ncRNA circuits may represent a promising strategy to simultaneously enhance antitumor immunity and limit EMT-mediated invasion and metastasis in GC. NK-associated ncRNA circuits appear predominantly permissive-they weaken immune surveillance and thereby facilitate the outgrowth of EMT-high tumor populations that are generated and maintained by upstream stromal and inflammatory cues (Section 4.1, Section 4.2 and Section 4.3), rather than serving as primary EMT triggers [111,112,113,114,115,116]. This interpretation supports tighter cross-reading of immune-evasion miRNA networks with the CAF/TAM/TAN-driven EMT ecosystem, especially when considering combination strategies that jointly target EMT signalling and immune checkpoints [111,113]. The key microenvironmental miRNA circuits discussed in Section 4.1, Section 4.2, Section 4.3 and Section 4.4 and their convergence on EMT programmes in GC are summarised in Figure 2.

Schematic overview of EMT in GC and representative miRNA axes operating in tumor cells and the tumor microenvironment (CAFs, TAMs, TANs/NETs, NK cells). EMT is associated with decreased E-cadherin and increased vimentin/N-cadherin. The figure highlights selected miRNA-mediated circuits linking stromal/immune components to EMT-related phenotypes, including immune-evasion-related NK dysfunction (e.g., PD-1/PD-L1-associated pathways) and NK depletion via ferroptosis-related signaling. The NK-cell module is organized into three subpanels: A (checkpoint/immune evasion), B (ferroptosis), and C (NK dysfunction). Arrows (↑/↓) indicate relative up- or downregulation of the indicated component in the shown context. Abbreviations: CAF-cancer-associated fibroblast; TAM-tumor-associated macrophage; TAN-tumor-associated neutrophil; NET-neutrophil extracellular trap; NK-natural killer.

### 4.5. Systemic Inflammatory Indices and Neutrophil-Derived Mediator FAM3C

Systemic inflammatory indices such as the neutrophil-to-lymphocyte ratio (NLR) and platelet-to-lymphocyte ratio (PLR) are simple, inexpensive markers that reflect the balance between pro-tumor inflammation and antitumor immunity. In early GC, Yasui et al. showed that pre-treatment NLR and PLR were significantly higher in patients with adenocarcinoma than in those with gastric adenoma and that elevated NLR was particularly associated with undifferentiated-type early GC and lymphovascular invasion, both features linked to EMT and metastatic potential [117]. Larger meta-analyses have confirmed that high NLR is associated with older age, male sex, greater depth of invasion, lymph-node involvement, and reduced 5-year overall survival in resected GC [118,119]. From a biological perspective, increased NLR reflects neutrophilia and relative lymphopenia, suggesting an immune landscape dominated by pro-tumor neutrophils and weakened adaptive responses-the same cellular configuration that, at the tissue level, supports EMT through IL-17A secretion, NET formation and neutrophil-tumor crosstalk described in the previous subsection.

A key soluble mediator that links neutrophil-rich inflammation to EMT in GC is family with sequence similarity 3, member C (FAM3C), also known as interleukin-like EMT inducer (ILEI). Yin et al. demonstrated that FAM3C protein is overexpressed in GC tissue compared with adjacent mucosa and that high FAM3C expression is significantly correlated with depth of invasion, lymph-node metastasis, advanced TNM stage, and reduced overall survival; in multivariable analysis, FAM3C overexpression emerged as an independent predictor of poor prognosis [120]. Immunohistochemistry showed that FAM3C expression clustered in invasive tumor fronts and in lesions with EMT-like morphology, supporting its role as a biomarker of EMT in GC [120]. These clinicopathological data are consistent with earlier experimental work identifying FAM3C/ILEI as a secreted factor that drives partial EMT and promotes metastatic traits in epithelial malignancies [121]. In co-culture and in vivo models, tumor cells secreted TGF-β1, which activated Smad2/3 signalling in neutrophils and upregulated FAM3C expression; FAM3C then acted back on tumor cells to activate JNK-ZEB1/Snail signalling, downregulate E-cadherin, and upregulate vimentin, ZEB1, and Snail, culminating in a robust EMT programme and increased lymph-node metastasis [122]. This FAM3C-centred loop provides a direct mechanistic bridge between neutrophil-dominated inflammation (high NLR), TGF-β-driven signalling, and EMT transcription factors that are themselves canonical targets of EMT-suppressive miRNAs such as the miR-200 and miR-34 families.

Although direct regulation of FAM3C by miRNAs has not yet been characterised in GC, evidence from other gastrointestinal tumors supports the concept that FAM3C is embedded within miRNA-controlled regulatory networks. In oesophageal squamous cell carcinoma, miR-574-3p was shown to target FAM3C and MAPK1, with miR-574-3p overexpression reducing proliferation and invasion, whereas low miR-574-3p levels were associated with poor prognosis and more aggressive disease [123].

Comprehensive reviews further highlight that FAM3C overexpression across multiple solid tumors is linked to EMT activation, cancer stem-cell traits, and metastatic spread, and that FAM3C expression is modulated by various oncogenic pathways and non-coding RNAs, including lncRNA-miRNA axes [124]. Together with the GC-specific data, these observations suggest that FAM3C functions as a cytokine-like EMT inducer at the intersection of neutrophil-driven inflammation and miRNA-regulated EMT transcriptional programmes.

From a translational standpoint, systemic inflammatory indices such as NLR and PLR, in combination with tissue or circulating measures of FAM3C, may serve as readouts of an EMT-permissive, neutrophil-rich microenvironment. In patients with elevated NLR and FAM3C expression, the EMT machinery in tumor cells is more likely to be engaged through JNK-ZEB1/Snail and TGF-β-Smad2/3 pathways. Targeting upstream inflammatory cues (e.g., TGF-β or neutrophil recruitment), neutralising FAM3C, or restoring the expression of EMT-suppressive miRNAs could therefore converge on the same downstream EMT nodes, offering a multi-layered strategy to mitigate invasion and lymph-node metastasis in GC.

Collectively, cross-referencing across the microenvironmental subsections suggests a tiered model of EMT regulation in GC. CAFs and TAMs most consistently exert dominant and sustained control by establishing a cytokine- and ECM-rich niche (TGF-β/IL-6/IL-8 signalling, exosomal miRNA exchange) that stabilises EMT transcription factors and mesenchymal traits in tumor cells [83,84,85,86,87,88,89,90,91,92,93,94,95,96,97,98,99,100,101,102,103]. In contrast, TAN-associated pathways often operate as context-dependent amplifiers, intensifying invasion through IL-17A-JAK2/STAT3 activation, NETs, and neutrophil-derived exosomal miRNAs in inflammation-enriched settings, which is coherently reflected by adverse systemic indices such as elevated NLR [104,105,106,107,108,109,110,117,118,119]. NK-cell dysfunction similarly functions as a permissive layer-by enabling immune escape, it allows EMT-high clones to persist and disseminate, reinforcing the clinical impact of stromal and inflammatory EMT drivers rather than initiating EMT uniformly across tumors [111,112,113,114,115,116]. Framing these components as dominant versus conditional helps reconcile why EMT-associated ncRNA signals are robust in some GC cohorts and experimental systems yet attenuated or heterogeneous in others, and it provides a rationale for stratified therapeutic strategies targeting both stromal EMT induction and immune escape.

This review synthesizes mechanistic and translational evidence indicating that miRNAs constitute an essential post-transcriptional layer modulating EMT programs in GC, thereby shaping invasion, metastatic competence, stemness-related traits, immune escape, and treatment resistance. Across the compiled evidence, miRNA effects repeatedly converge on a limited set of “core EMT hubs”, particularly the ZEB/E-cadherin module, TGF-β-driven EMT circuits, and Wnt/β-catenin activity, suggesting that phenotypically diverse miRNA candidates frequently drive overlapping outputs through shared pathway-level dependencies rather than fully distinct mechanisms [14,57,60,61].

### 4.6. Hierarchy of Evidence and Robustness of EMT-miRNA Claims

A clearer hierarchy of evidence is necessary to interpret the EMT-miRNA literature in GC, because the strength of support varies substantially between candidates and study designs. Much of the mechanistic evidence originates from in vitro gain-/loss-of-function experiments in established GC cell lines and often relies on a minimal EMT marker panel (typically E-cadherin with one or two mesenchymal markers), rather than layered phenotyping that captures plasticity, invasion dynamics, or spatial context [60,63]. In vivo evidence (e.g., xenografts) strengthens mechanistic claims but still only partially captures the complexity of human GC, given immune deficiencies in many models and limited representation of stromal/immune crosstalk [29,82]. Evidence becomes stronger when multiple layers (human + functional + in vivo) align in a coherent direction. A representative example is miR-34a, where the human tissue signal (61 paired samples) is paired with mechanistic lncRNA-mediated epigenetic repression, EMT marker reversal, and an experimental metastasis readout in vivo (tail vein model, *n* = 7 mice), collectively supporting a biologically plausible EMT-suppressive role [57]. Nevertheless, even such comparatively well-supported candidates still require external cohort validation and models that capture organ-specific dissemination and microenvironmental constraints more faithfully.

### 4.7. Context Dependence, Conflicting Reports, and EMT Plasticity

A central unresolved issue in EMT biology is that EMT is not a binary epithelial-to-mesenchymal switch but rather a continuum of intermediate, hybrid, and reversible states, including MET-like re-epithelialization during metastatic colonization [18,125]. This plasticity introduces interpretive variability: identical miRNA perturbations can yield different phenotypes depending on baseline pathway wiring, stromal cytokine context, and the stage of dissemination. For example, miRNAs that regulate canonical EMT nodes may show strong cell-autonomous effects in vitro, whereas in vivo and in human tumors EMT is frequently co-driven by microenvironmental signals (e.g., TGF-β-rich niches), potentially altering both the magnitude and directionality of observed associations [60,79]. An additional driver of conflicting reports is compartment mixing: bulk-tissue miRNA signals can reflect epithelial tumor cells as well as stromal and immune components that actively shape EMT programs. Without controlling for tumor purity or microenvironmental composition, tumor-intrinsic interpretation of miRNA signatures may be overstated, contributing to inconsistent “oncogenic vs. suppressive” classification across settings. These limitations argue for studies that explicitly resolve epithelial versus stromal compartments and assess EMT states using broader phenotyping frameworks rather than minimal marker switching alone.

### 4.8. Cohort Consistency Across Populations and Experimental Systems

A persistent limitation is the incomplete appraisal of consistency across cohorts, ethnic populations, and experimental systems. Many human EMT-miRNA studies in GC are single-center and modest in size, limiting generalizability and increasing the risk of context-specific findings. This is evident in multiple candidates where clinical signals rely on small cohorts (e.g., miR-204: paired tissues *n* = 24; metastasis subgroup *n* = 14) [60], while some circulating biomarker studies provide larger sample frames yet still represent single-institute designs (e.g., plasma miR-101: GC *n* = 128 vs. controls *n* = 80) [62]. Model dependence further complicates consistency: distinct GC cell lines differ in baseline EMT propensity, pathway dependencies (TGF-β responsiveness, Wnt signaling status), and genetic backgrounds, which can shift sensitivity to miRNA perturbation and weaken reproducibility across laboratories. Therefore, robust translational claims should ideally be supported across multiple models, including complementary systems such as organoids, and validated in independent human cohorts stratified by clinicopathological context and subtype.

### 4.9. Methodological Weaknesses and Standardization Gaps

Methodological heterogeneity substantially limits cross-study comparability and weakens translational inference. Variability in specimen handling, RNA extraction, measurement platforms (qPCR vs. sequencing), normalization strategies, and analytical pipelines can distort both direction and effect size of miRNA-EMT associations, particularly for circulating miRNAs [126,127]. Pre-analytical factors (serum vs. plasma choice, hemolysis, centrifugation protocols, freeze-thaw cycles, storage time) can introduce large non-biological variance that may exceed the biological signal, undermining reproducibility [126]. Normalization is a recurring vulnerability; commonly used endogenous controls such as U6 are not consistently stable in circulation, which can lead to systematic bias and false signatures [127]. These weaknesses are directly relevant to the EMT-miRNA field because many candidates are proposed as biomarkers, yet methodological non-standardization makes it difficult to reconcile results across laboratories or develop clinically deployable assays.

### 4.10. Translational Barriers: Pleiotropy, Off-Target Effects, and Delivery Constraints

MiRNA-based therapeutics remain conceptually attractive because miRNAs can reprogram networks rather than single targets, but this same property increases the risk of pleiotropy-driven off-target effects and unintended pathway rewiring [128]. Delivery remains a major bottleneck: miRNA mimics and inhibitors require protection from degradation, tumor-selective biodistribution, adequate cellular uptake, and appropriate intracellular release [129]. A key translational cautionary example is MRX34 (liposomal miR-34a mimic), which demonstrated feasibility but was terminated early due to severe immune-mediated toxicities, underscoring that safety and immunogenicity can limit miRNA therapeutic development despite strong mechanistic rationale [128].

Finally, EMT reversibility complicates endpoint selection and therapeutic timing. Since dissemination can involve transient partial EMT states and metastatic establishment may require MET-like re-epithelialization, indiscriminate or temporally mismatched EMT targeting may yield limited benefit or paradoxical effects [125]. Translational approaches may therefore need dynamic biomarkers capable of capturing epithelial-mesenchymal plasticity longitudinally and should be integrated with upstream pathway-level inhibitors where appropriate.

### 4.11. Future Directions

Future progress requires (1) multi-cohort, multi-ethnic validation, (2) standardized pre-analytical and analytical pipelines, and (3) stronger model triangulation (multiple GC subtypes, organoid-based systems, and in vivo confirmation). Methodologically, studies proposing biomarkers should adopt robust quality control and validated normalization strategies to ensure inter-laboratory comparability. Biologically, prioritizing candidates that reproducibly converge on core EMT hubs across complementary systems-and remain consistent after accounting for microenvironmental composition-will strengthen interpretability and improve the translational trajectory of EMT-miRNA findings in GC.

## 5. Conclusions

EMT is a central determinant of invasion, metastasis, immuno-evasion, and therapy resistance in GC, and miRNAs occupy a pivotal position within the regulatory networks that govern this process. The evidence synthesized in this review indicates that GC EMT is coordinated by a complex interplay between downregulated tumor-suppressive miRNAs and upregulated oncogenic miRNAs, acting both within tumor cells and across the tumor microenvironment. CAFs, TAMs, TANs, NK cells, and systemic inflammatory mediators contribute additional ncRNA-centred layers of control, creating feed-forward circuits that stabilize EMT and accelerate disease progression. The miRNAs summarized in Table 1 have clear potential as diagnostic, prognostic, and predictive biomarkers, as well as candidate therapeutic targets aimed at reversing EMT and improving clinical outcomes. At the same time, substantial work remains to validate these candidates in large, prospective cohorts, to integrate them with molecular subtyping frameworks, and to translate them into safe, effective miRNA-based interventions. A deeper understanding of how EMT-related miRNAs coordinate crosstalk between tumor cells, stroma, and immune compartments may ultimately enable more precise, pathway-directed therapies that simultaneously curb metastasis, restore chemosensitivity, and enhance anti-tumor immunity in patients with GC.

## Figures and Tables

**Table 1:** Downregulated and upregulated miRNAs involved in gastric cancer EMT.

**Downregulated EMT-Related miRNAs in Gastric Cancer**						
**miRNA**	**Study Type**	**Sample Type**	**miRNA Detection Platform**	**Normalization**	**Key Findings**	**Limitations**
miR-34a [57]	Human + in vitro + in vivo	Human tissues: matched tumor vs. adjacent non-tumor from 61 GC patients (no neoadjuvant therapy). In vivo: tail vein model using BGC-823 cells injected into 7 athymic male mice.	qRT-PCR (SYBR, PrimeScript RT + SYBR Premix Ex Taq; ABI platform)	U6 (miR-34a); GAPDH (HOTAIR)	miR-34a is downregulated in GC tissues and negatively correlated with HOTAIR; HOTAIR knockdown increases miR-34a (~4.2-fold), reverses EMT (E-cad↑, N-cad/VIM↓), and reduces migration/invasion. Mechanistically, HOTAIR recruits PRC2 (EZH2/SUZ12) to enforce H3K27me3 at the miR-34a promoter; restoring miR-34a suppresses c-Met/Snail and EMT; sh-HOTAIR reduces lung metastases in vivo.	Single-center human cohort; no independent external clinical validation cohort; tmetastasis assessed mainly via experimental tail-vein model
miR-200 family (miR-200a/200b/200c/141/429) [14]	In vitro + in vivo + in silico (multiplex CRISPR/Cas9 complete miR-200 family KO in GC cell lines + xenografts; TCGA/ACRG transcriptomic comparison)	In vitro: human GC cell lines AGS (single-cell cloning; 39 clones screened) + MKN28 validation. In vivo xenografts: NSG mice with *n* = 4 per group (A-NTC vs. A-31 FKO vs. A-26 residual miR-200).	qPCR for miR-200 family validation (proxy screening: miR-200c + miR-429; extended panel); RNA-seq (steady-state transcriptome); WGS used to assess CRISPR off-targets	miRNA qPCR used miR-21 as control (explicitly stated for the miR-200 panel)	Complete miR-200 loss induced EMT-like changes (junction/cytoskeleton disruption; EMT genes ↑) but simultaneously triggered strong senescence phenotype (↑SA-β-Gal, G1/S arrest, ↑p21/p53 axis), aberrant metabolic reprogramming, and SASP/TGF-β + TNF-α pathway enrichment. In vivo, miR-200 FKO clones formed slower-growing xenografts and showed stromal recruitment signatures; “low-miR-200” TCGA tumors aligned transcriptomically with ACRG EMT subtype.	Mechanistic preclinical design (CRISPR editing) → potential confounding from DSB/p53 activation (authors discuss); NSG mice immunodeficient, limiting immune-TME inference; normalization/qPCR workflow not fully detailed in main text
miR-200b [58]	Humans + in vitro	Human cohort: 60 GC resection patients (tumor vs. “normal control” tissues). Patients stratified into high miR-200b (*n* = 29) vs. low miR-200b (*n* = 31) for clinicopathological correlations. In vitro: GC cell lines MGC-803 + BGC-823 vs. normal GES-1; HEK-293T for luciferase assays.	qRT-PCR for miR-200b (±mRNA targets); WB for EMT/NRG1 axis; Transwell migration/invasion; dual-luciferase (NRG1 3′UTR)	qRT-PCR normalized to U6 (miRNA) and GAPDH (mRNA/protein WB control); relative expression via 2^−ΔΔCt^	miR-200b was reduced in GC tissues/cells; ectopic miR-200b suppressed migration/invasion, promoted epithelial phenotype (↑E-cadherin, ↓vimentin), and directly targeted NRG1 (luciferase validation), consistent with inhibition of NRG1-ERBB2/ERBB3 signaling. Clinically, lower miR-200b associated with adverse clinicopathologic features and worse survival (as reported by authors).	Single-centre cohort; no in vivo validation; survival analysis appears largely univariate (risk of confounding); cut-off defined by mean expression; qPCR-only quantification (no orthogonal assay like ISH in cohort).
miR-148a [59]	Human + in vitro	Human: 60 GC patients, paired tumor + adjacent non-tumor mucosa from all cases (gastrectomy; no pre-op therapy stated). In vitro: MKN-45 (stable miR-148a overexpression via lentivirus; inhibitor experiments also performed); HEK-293T (luciferase + lentiviral packaging).	TaqMan miRNA Assay (Applied Biosystems, Foster, CA, USA) for miR-148a qRT-PCR; mRNAs (SMAD2, E-cadherin, vimentin) by SYBR Green qRT-PCR	miR-148a: RNU48; mRNAs: GAPDH; WB loading control: β-actin	miR-148a was significantly downregulated in GC tissues vs. matched non-tumor mucosa (54/60 tumors showed downregulation). Low miR-148a was associated with adverse clinicopathological features (reported: lymph node metastasis/N stage/blood vessel invasion). Stable miR-148a overexpression reduced migration/invasion and reversed EMT markers (E-cadherin↑, vimentin↓). SMAD2 was validated as a direct functional target (luciferase + reduced SMAD2 mRNA/protein).	Single-centre cohort (*n* = 60); no in vivo metastasis/therapy validation; EMT readouts mainly limited to E-cadherin/vimentin; clinical outcomes (e.g., survival) not reported in the excerpted sections.
miR-204 [60]	Human + in vitro (TGF-β-induced EMT model + functional assays)	Human: 24 matched GC tissues (tumor + adjacent non-tumor mucosa; resection; no pre-op chemo/RT). Subgroup comparison reported: metastasis group *n* = 14 vs. no-metastasis group (remaining cases). In vitro: AGS + BGC GC cell lines; HEK293/HEK293T for luciferase; EMT-like transformation induced by TGF-β1 (10 ng/mL, 21 days) to generate AGS-T/BGC-T.	miRNA detection by poly(A) tailing + SYBR Green qRT-PCR (miR-204); initial screening also included conventional RT-PCR. mRNAs by SYBR Green qRT-PCR; luciferase reporter assay for SIRT1 3′UTR	miR-204: U6 internal control; mRNAs: GAPDH; luciferase: pRL-TK Renilla as transfection control; WB loading control: β-actin	miR-204 was downregulated in GC tissues vs. matched normal mucosa and was lower in the reported metastasis subgroup. miR-204 directly targeted SIRT1 (WT vs. MUT 3′UTR luciferase). miR-204 restoration reduced invasion, reversed EMT markers (E-cadherin↑, vimentin↓) in TGF-β-transformed cells, and reduced anoikis resistance (Annexin V/PI on poly-HEMA). SIRT1 knockdown phenocopied miR-204 effects; authors link pathway to LKB1 regulation.	Small human cohort (*n* = 24); metastasis subgroup definition is cohort-specific (no external validation); experiments limited to two cell lines and in vitro EMT induction; no in vivo validation of metastasis or therapeutic delivery.
miR-30a [23,61]	Humans + in vitro + in vivo	Human tissues: primary GC *n* = 55 (tumor + surrounding normal mucosa); RUNX3 protein quantified in subset *n* = 25. Cell lines: AGS, BGC-823, SGC-7901, KATOIII, GES-1. In vivo: nude mice tail-vein metastasis model using stable BGC-823 (6 × 10^6^ cells/mouse)	miRNA profiling by miRCURY LNA Array v16.0 (scanned with Axon GenePix 4000B, Molecular Devices, Inc., Sunnyvale, CA, USA); qRT-PCR for miR-30a; luciferase reporter (vimentin 3′UTR WT/MUT); ChIP-PCR for RUNX3 binding at miR-30a promoter; Western blot	RUNX3 protein in tissues expressed as RUNX3/GAPDH (WB). miRNA qRT-PCR normalization not explicitly stated in the accessible text.	miR-30a directly targeted vimentin 3′UTR and lowered vimentin protein; miR-30a inhibitor reversed RUNX3-driven suppression of invasion and vimentin downregulation. Clinically, RUNX3 was reduced in 35/55 (63.6%) tumors; RUNX3 correlated positively with miR-30a and negatively with vimentin.	Mouse experiment group size not reported; patient cohort used mainly for correlation (no survival/outcome modeling); single setting; miRNA qRT-PCR reference control not clearly described, which affects reproducibility.
	Humans + in vitro	Human: advanced GC patients *n* = 20 → chemo-sensitive (*n* = 13) vs. chemo-resistant (*n* = 7). Cells: cisplatin-resistant SGC-7901/DDP vs. parental SGC-7901; functional rescue with miR-30a mimic/inhibitor.	TaqMan miRNA assays (Applied Biosystems)	2^−ΔΔCt^ approach reported; endogenous control for miRNA qPCR not specified in the extracted text	miR-30a is reduced in cisplatin resistance and its restoration increases cisplatin sensitivity. Mechanistically, miR-30a targets YAP1, reducing downstream pro-survival/EMT-associated behavior, and shifting cells toward a more chemo-responsive phenotype.	Small clinical cohort (especially resistant group *n* = 7). Predominantly one resistant cell-line model, and no in vivo validation reported here. Missing/unclear reporting for miRNA qPCR endogenous control reduces methodological transparency.
miR-101 [62]	Humans + in vitro	Plasma: GC *n* = 128 vs. healthy controls *n* = 80; Exosomes: GC *n* = 4 vs. healthy *n* = 4; Tissue: GC *n* = 8 vs. normal mucosa *n* = 8; GC cell lines (multiple; functional work mainly in MKN45)	TaqMan qRT-PCR (Applied Biosystems) in plasma/exosomes/tissue/cells; ROC analysis; functional assays (CCK-8 growth, colony formation, FACS cell cycle/apoptosis, Transwell migration/invasion); Western blot	Plasma qRT-PCR normalized using cel-miR-39 spike-in (2^−ΔΔCt^); tissue/cells normalized to U6 (RNU6B)	Plasma miR-101 was significantly downregulated in GC and associated with advanced T stage, advanced TNM stage, and peritoneal metastasis; low plasma miR-101 predicted worse prognosis (HR ~3.07 independent of TNM stage). Diagnostic performance: AUC ~0.740, sens 56.3%, spec 82.5% (cut-off 8.64). Functionally, miR-101 restoration induced apoptosis via MCL1 suppression and reduced migration/invasion via ZEB1 downregulation (with EMT shift, incl. ↑E-cadherin).	Clinical part described as relatively small retrospective single-institute cohort; limited exosome/tissue subset sizes; no independent external validation cohort.
miR-218 [63]	In vitro	GC cell lines SGC7901, BGC823 vs. normal gastric epithelial GES-1; functional assays after miR-218 mimic/inhibitor transfection; experiments performed ≥ triplicate	qRT-PCR (SYBR Green) on ABI Prism 7500; dual-luciferase reporter assay (WASF3 3′UTR WT/MUT); Western blot	miR-218 normalized to U6; WASF3 mRNA normalized to GAPDH; WB control β-actin; luciferase internal Renilla (pRL-TK)	miR-218 was lower in GC cell lines, while WASF3 was higher. miR-218 overexpression suppressed proliferation and migration and shifted EMT markers toward epithelial phenotype (↑E-cadherin; ↓N-cadherin/vimentin/TWIST1). WASF3 confirmed as a direct target (3′UTR reporter); restoring WASF3 partially rescued miR-218 effects.	No human clinical cohort and no animal validation; limited number of cell lines; mainly short-term functional assays (e.g., MTT, scratch); generalizability/clinical relevance not assessed.
miR-26a [64]	Humans + in vitro + in vivo	Human tissues: 40 paired GC + adjacent non-tumor tissues (qRT-PCR). Tissue microarrays: 126 GC + 41 adjacent normal (ISH). In vivo: subcutaneous xenograft *n* = 5/group; tail-vein metastasis model *n* = 6/group.	Expression: qRT-PCR (miR-26a), in situ hybridization (ISH) on TMA. Functional assays: proliferation, colony formation, migration/invasion (in vitro). Target validation: luciferase reporter, Western blot for FGF9. In vivo tumor growth and metastasis models.	qRT-PCR for miRNA normalized to U6 snRNA. ISH used U6 as positive control.	miR-26a was downregulated in GC vs. adjacent non-tumor tissues and associated with advanced clinical features. Overexpression of miR-26a suppressed proliferation, migration, invasion and metastasis. FGF9 identified and validated as a functional target: miR-26a decreased FGF9 protein and inhibited luciferase activity of FGF9 3′UTR reporter. In vivo, miR-26a reduced tumor growth (*n* = 5/group) and tail-vein metastasis burden (*n* = 6/group).	Moderate-sized clinical cohort (qRT-PCR *n* = 40; ISH cohort larger but still single setting). Mainly preclinical functional endpoints; broader translational robustness (independent cohorts/circulating miRNA) not assessed. Mechanistic axis centered on FGF9; other potential targets not deeply explored.
miR-486-5p [65]	In vitro	Cell-line exosomes: GC9811 vs. GC9811-P (peritoneal metastatic subline). Recipient cells: HMrSV5 (human peritoneal mesothelial cells). No human samples.	Exosome isolation (ExoQuick-TC). Exosome validation: TEM, NTA (NanoSight NS300) (Malvern Panalytical, Malvern, UK), WB (CD9, CD63). miRNA profiling: Agilent Human miRNA microarray. Validation: qRT-PCR.	Microarray normalized in GeneSpring (quantile normalization + baseline transformation). qRT-PCR: RUN6-1 (U6) (miRNA) and GAPDH (mRNA).	PM-derived exosomes (GC9811-P-Exo) induced stronger EMT-like changes in mesothelial cells vs. control exosomes. miR-486-5p and miR-132-3p were downregulated in PM-Exo, while miR-132-5p was upregulated. miR-486-5p overexpression attenuated EMT-related phenotype (e.g., α-SMA changes) and reduced expression of candidate downstream molecules (SMAD2, CDK4, ACTR3).	Entirely cell-line based (no patient/clinical validation). Targeting relationships mainly associative (no definitive luciferase confirmation of direct binding for proposed targets). Exosome isolation via precipitation kit may increase co-isolation/impurities vs. ultracentrifugation.
miR-16-5p [66]	Human + in vitro + in vivo	Human qRT-PCR cohort: 50 paired GC + matched paracancer tissues (fresh; no neoadjuvant therapy). IHC cohort: 57 GC patients. Cell lines: GES-1 + GC lines (BGC-823, HGC-27, MKN-45, SGC-7901, MGC-803, AGS). In vivo xenograft: MGC-803 (sh-circPGD/sh-ABL2/control), *n* = 4/group, 2 × 10^6^ cells. In vivo metastasis: tail-vein BGC-823 (GFP-labelled), *n* = 4/group, 2 × 10^6^ cells.	qRT-PCR (ABI Step One Plus) (Thermo Fisher Scientific, Waltham, MA, USA), RNase R validation, nuclear/cytoplasmic fractionation + RNA-FISH. Dual-luciferase reporter (circPGD-miR-16-5p & miR-16-5p-ABL2). Western blot / IF; functional assays (Transwell, wound healing, colony formation, CCK-8, apoptosis). LC-MS/MS to confirm PGD-219aa peptide.	qRT-PCR quantification: 2^−ΔΔCt^, endogenous reference GAPDH	circPGD sponges miR-16-5p, releasing repression of ABL2, with downstream involvement of SMAD2/3 and YAP signaling. circPGD also encodes PGD-219aa, which enhances migration/growth and supports EMT-related protein changes. In vivo: circPGD/ABL2 knockdown reduces xenograft growth; circPGD overexpression increases metastatic aggressiveness.	Single-center patient cohorts (50 qRT-PCR; 57 IHC) and small mouse group size (*n* = 4/group).
miR-375 [67]	Human + in vitro + in vivo	Human tissue microarray: 17 primary GC, 12 adjacent tissues, 16 metastatic GC, 5 normal gastric mucosa. Cell lines: HGC-27, MGC-803, BGC-823, SGC-7901. In vivo: BALB/c nude mice; tail-vein lung metastasis model (*n* = 3/group, 2 × 10^6^ SGC-7901 cells). Tumor initiation/limiting dilution: subcutaneous injections (1 × 10^7^/5 × 10^6^/2.5 × 10^6^ cells; mice number not specified).	qRT-PCR for miR-375 & mRNAs; Affymetrix Clariom™ D Assay 2.0 gene expression microarray (SGC-7901 ± miR-375 overexpression); IHC (SLC7A11), RNA-FISH (miR-375) on tissue chip	miRNA qRT-PCR: U6; GAPDH	miR-375 directly targets SLC7A11, promoting ferroptosis; in vivo, miR-375 overexpression reduced lung metastasis burden in the tail-vein model	Authors explicitly note limited tissue sample size, and discuss that their observation about SLC7A11/metastasis may appear contradictory to the conventional view; mouse numbers were small in the metastasis model (*n* = 3/group).
miR-506/miR-506-5p [68,69]	Humans + in vitro	Human tumors: 141 GC tumors (qRT-PCR for miR-506). Groups: low (*n* = 85) vs. high (*n* = 56). Subgroup without peritoneal metastasis: low (*n* = 71) vs. high (*n* = 47). IHC subset: 39 GC patients (SNAI2 protein). Cell lines: MKN7, MKN45 (pre-miR-506 experiments); additional lines referenced for baseline miR-506/SNAI2 comparison.	qRT-PCR for miR-506 and mRNA targets; luciferase reporter assay for SNAI2 3′UTR binding; WB/qPCR for E-cadherin; functional assays for proliferation/migration.	Not explicitly detailed in the main text for miR-506 qRT-PCR. For mechanistic assays: luciferase readouts vs. negative controls; E-cadherin assessed at mRNA/protein level post-transfection.	Low miR-506 expression associated with poorer differentiation and worse overall survival. In multivariate analysis, miR-506 was an independent prognostic factor (relative risk 1.78, 95% CI 1.00-3.30, *p* = 0.049). Mechanistically, miR-506 directly represses SNAI2, and miR-506 overexpression increases E-cadherin (mRNA and protein), supporting EMT inhibition.	Human data are observational (expression-outcome associations). No in vivo validation. IHC performed only in a subset (*n* = 39). Some experimental protocol specifics (incl. normalization details) are not fully shown in the main text.
	Humans + in vitro + in silico	Human tissues: 46 paired gastric adenocarcinoma vs. matched para-cancer tissues (qRT-PCR for LINC01232 and miR-506-5p). Cell lines (expression): GES-1 vs. AGS, BGC-823, HGC-27, SGC-7901. Functional assays: mainly AGS + SGC-7901. 293T used for luciferase binding assays.	qRT-PCR (SYBR, Bio-Rad CTF100) (Bio-Rad Laboratories, Inc., Hercules, CA, USA); WB for EMT proteins (E-cadherin/vimentin) and PAK1; transwell migration, wound healing; dual-luciferase reporter assays for LINC01232-miR-506-5p and miR-506-5p-PAK1 interactions.	qRT-PCR normalization: U6 for LINC01232 + miR-506-5p, GAPDH for PAK1; luciferase normalized to Renilla. WB normalized to GAPDH.	LINC01232 is upregulated in GC (TCGA + 46 paired samples) and acts as a competitive endogenous RNA binding miR-506-5p (luciferase confirmation). miR-506-5p is downregulated in GC tissues/cell lines and suppresses GC cell proliferation/migration/EMT-like phenotypes; PAK1 validated as a miR-506-5p target, and PAK1 knockdown attenuates LINC01232-driven migration.	No in vivo model. Small patient sample (*n* = 46).
miR-338-3p [70]	Humans + in vitro	Human tissues: 20 paired GC vs. adjacent non-cancerous tissues (qRT-PCR; also ISH/IHC performed on tissue sections). Staging: I-II (*n* = 9) vs. III-IV (*n* = 11). Cell lines: AGS, MKN-28 (main functional assays); additional GC lines tested for baseline expression; HEK-293T for luciferase assays.	qRT-PCR (SYBR-based), ISH (LNA probes) for miR-338-3p localization, dual-luciferase reporter assays (ZEB2/MACC1 3′UTR), WB for EMT markers; wound healing, Transwell, 3D culture.	qRT-PCR normalized to snRNA U6 (miRNA) and β-actin (mRNA); 2^−ΔΔCt^ method.	miR-338-3p is reduced in GC tissues and lower in advanced stage. miR-338-3p overexpression suppressed migration/invasion and shifted EMT markers toward epithelial phenotype (E-cadherin↑; mesenchymal markers↓). Mechanistically, miR-338-3p directly targets ZEB2 and MACC1 (luciferase validation), implicating inhibition of EMT-driving circuitry.	Small clinical cohort (*n* = 20), limited power for clinicopathologic stratification. No in vivo model. Mostly 2 main GC cell lines used for functional validation.
miR-2392 [27]	Human + in vitro + in vivo	Human tissues: tissue microarrays with 84 paired GC + adjacent normal tissues (ISH for miR-2392; IHC for MAML3/WHSC1; clinicopath + survival analyses). Normal gastric tissues also collected from gastroscopy patients. In vitro: GC cell lines AGS, SGC7901, BGC823, GC9811, MKN45 + nonmalignant GES; HEK293T for luciferase. In vivo metastasis: tail-vein injection of BGC823-luc cells; nude mice *n* = 6/group (miR-2392 agomir vs. negative control), lung metastasis assessed at 4 weeks by IVIS + H&E.	qPCR (LightCycler 480 (Roche, CHE); SYBR Green master mix) for miR-2392 + target genes; ISH (miRCURY LNA probe) for miR-2392 on TMAs; IHC for MAML3/WHSC1; dual-luciferase (psiCHECK-2) for MAML3/WHSC1 3′UTRs; WB for EMT/TF markers	miR-2392 qPCR normalized to U6; mRNAs normalized to β-actin; WB loading control β-actin; luciferase normalized to Renilla	miR-2392 was downregulated in GC tissues and cell lines, lower in stage III-IV vs. stage I-II, and associated with more aggressive clinicopathologic features. Low miR-2392 expression predicted worse OS and remained an independent prognostic factor in Cox analysis. Functionally, miR-2392 inhibited migration/invasion (no major effect on proliferation/cell cycle). Mechanism: miR-2392 directly suppressed MAML3 and WHSC1, leading to reduced Slug/Twist1, increased E-cadherin, decreased vimentin, and EMT inhibition; knockdown of MAML3/WHSC1 phenocopied miR-2392 effects. In vivo, miR-2392 overexpression markedly reduced lung metastasis after tail-vein injection.	Single-center tissue resource (*n* = 84 pairs) and a tail-vein metastasis model (non-orthotopic). Mechanistic validation largely in selected GC cell lines; therapeutic feasibility/delivery not addressed beyond agomir transfection prior to injection.
**Upregulated EMT-Related miRNAs in Gastric Cancer**						
**miRNA**	**Study Type**	**Sample Type**	**miRNA Detection Platform**	**Normalization**	**Key Findings**	**Limitations**
miR-17-5p [71,72]	Human + in vitro + in vivo	Human tissues: paired gastric tumor vs. adjacent normal from 28 patients (miR-17-5p higher in 18/28 tumors). Cells: SGC7901, MKN28 (stable lenti-miR-17-5p overexpression + inhibitor). In vivo: BALB/c nude mice xenografts, 6 mice/group (lenti-miR-17-5p vs. lenti-NC).	qRT-PCR for miR-17-5p; luciferase reporter (SOCS6 3′UTR WT/MUT); Western blot/IHC, MTT + colony assays; in vivo IVIS bioluminescence imaging.	U6 (miRNA); β-actin (SOCS6 mRNA).	miR-17-5p was overexpressed in GC tissues vs. adjacent normal. Overexpression increased proliferation (MTT/colony) and enhanced xenograft tumorigenicity. SOCS6 confirmed as direct target (3′UTR luciferase; SOCS6 protein down with miR-17-5p). Restoring SOCS6 (without 3′UTR) attenuated miR-17-5p pro-proliferative effects.	Human cohort relatively small (*n* = 28) and clinicopathologic associations were limited. Focused mainly on proliferation, not a full metastasis program. Single-center tissue set; limited independent external validation.
	Bioinformatics + in vitro + in vivo	TCGA/UALCAN analyses only (no independent patient tissue validation). Cells: SGC-7901, MGC-803, AGS (stemness assays mainly emphasize MGC-803). In vivo: subcutaneous xenografts in 18 male BALB/c nude mice (4 weeks old); group allocation NR.	RT-PCR (SYBR-green); dual-luciferase for promoter/3′UTR assays; ChIP (MKL-1 binding to promoters), RIP/RNA pull-down; WB/IHC; sphere formation + drug resistance assays.	U6 (miRNA); GAPDH (mRNA).	TCGA analysis suggested miR-17-5p and MKL-1 are increased in GC, with worse prognosis in high-expression groups (database-level). Mechanistically, MKL-1 activated the promoters of CD44, EpCAM, and miR-17, promoting stem-like traits. miR-17-5p targeted MKL-1 3′UTR (luciferase + RIP/pull-down), inhibiting MKL-1 expression. In xenografts, miR-17-5p/MKL-1 modulation supported effects on tumor stemness markers (CD44/EpCAM) and tumor growth-related outputs.	No primary human cohort (clinical part relies on public databases). Xenograft group sizes/design not clearly specified beyond total *n* = 18. Broad mechanistic model with many assays may limit reproducibility without external validation.
miR-106b-5p [73]	Human + in vitro + in vivo	Human tissues: metastatic vs. non-metastatic EGC tumor tissues (sample size NR). Cells: AGS gastric cancer cells. In vivo: AGS xenograft BALB/c nude mice; 5 groups (Control/NC/GLPG0634/miR-106b inhibitor/inhibitor + GLPG0634), n per group NR.	qRT-PCR (TaqMan miRNA assay) + FISH for tissues; qRT-PCR in cells.	U6 reference gene (2^−ΔΔCt^).	miR-106b was higher in metastatic EGC tissues (~2-fold vs. non-metastatic). miR-106b mimics increased migration/invasion and EMT-related proteins; inhibitor increased apoptosis. ALEX1 validated as a direct target (luciferase); miR-106b inhibition reduced pJAK1/pSTAT3, and JAK1 overexpression rescued apoptosis effects. In xenografts, miR-106b inhibition reduced tumor growth, enhanced by GLPG0634.	Clinical tissue n not reported; xenograft group sizes not reported. Functional work largely in one GC cell line (AGS). No external clinical validation cohort/prognostic endpoint testing reported.
miR-23a [74]	Human + in vitro + in vivo	Clinical samples: expression correlation reported using 38 stage I GC patients (miR-23a by RT-PCR + PTEN by IHC). DFS analysis reported for stage I cohort with follow-up data (*n* = 91, stages II-III not analyzed due to limited numbers). In vitro: human GC AGS, SGC-7901, normal gastric GES-1. In vivo: (1) subcutaneous tumor-bearing study in C57BL/6 mice, 5 mice/group (murine gastric adenocarcinoma cells; intratumoral miR-23a precursor injections); (2) intraperitoneal model reported in BALB/c nu/nu mice, *n* = 3/group; (3) subcutaneous xenograft model reported in nude mice, *n* = 10/group using transfected cells (as described in figure legend).	TaqMan miRNA qRT-PCR on 7900HT Real-Time PCR System (Applied Biosystems, Foster, CA, USA); dual-luciferase PTEN 3′UTR; WB/IHC for PTEN and pathway markers; functional Transwell invasion assays.	miR-23a qRT-PCR normalized to RNU6B (U6); WB loading control actin; luciferase normalized to Renilla.	miR-23a inhibition reduced GC cell migration/invasion. miR-23a directly targeted PTEN (3′UTR luciferase) and PTEN loss promoted invasion. miR-23a-mediated PTEN suppression activated AKT/ERK signaling and EMT-associated changes, and was associated with enhanced xenograft growth in vivo. Clinically, high miR-23a with low PTEN was proposed as a risk factor; PTEN status associated with DFS in stage I subgroup (as reported).	Clinical analyses restricted to stage I and small correlation cohort (*n* = 38); DFS analysis appears limited and not extended to stages II-III; multiple animal experiments reported with differing models/cell types and some internal inconsistencies in descriptions (must be interpreted cautiously); no external cohort validation.
miR-130a-3p [75]	Human + in vitro + in vivo	Human tissues: 45 paired gastric tumor vs. adjacent normal tissues (same patients; *n* = 45 vs. *n* = 45). In vitro: GC cell lines HGC-27, MKN45, AGS (functional assays mainly in MKN45 & AGS), normal gastric GES-1. In vivo: BALB/c nude mouse subcutaneous xenografts using MKN45 (5 × 10^5^ cells); antagomiR vs antagomiR-NC treatment every 48 h.	RT-qPCR (SYBR-based) for miR-130a-3p and GCNT4; dual-luciferase (GCNT4 3′UTR); RNA pull-down; WB for TGF-β1/SMAD3 signaling.	miR-130a-3p normalized to U6; GCNT4 normalized to GAPDH; 2^−ΔΔCt^ used; dual-luciferase reported as firefly/Renilla.	miR-130a-3p was upregulated in GC tissues/cell lines, while GCNT4 was downregulated. miR-130a-3p enhanced proliferation/migration/invasion in vitro and promoted tumor growth in xenografts; GCNT4 overexpression counteracted these effects. Mechanistically, miR-130a-3p directly suppressed GCNT4, with downstream activation of TGF-β1/SMAD3 pathway (↑p-SMAD3).	Single-center cohort (*n* = 45); in vivo group size not reported; outcomes focus on growth/motility signaling (no metastasis model); heavy reliance on RT-qPCR/WB (limited orthogonal clinical validation).
miR-196a-5p [76]	Human + in vitro	In vitro: CD44(+) vs. CD44(−) cells sorted by FACS from SNU-5 and BGC-823 (microarray performed on CD44(+) vs. CD44(−) SNU-5). Human tissue: 95 paired GC vs. adjacent non-tumorous tissues assessed by IHC for SMAD4 (clinical association analyses for SMAD4).	miRNA microarray: Affymetrix GeneChip miRNA 2.0. Validation: Bulge-Loop miRNA qRT-PCR (ABI7500); SMAD4 mRNA by qRT-PCR; dual-luciferase 3′UTR assays.	miRNA qRT-PCR normalized to U6; mRNA qRT-PCR normalized to GAPDH.	miR-196a-5p was upregulated in CD44(+) stem-like GC cells and promoted sphere formation and invasion. miR-196a-5p directly suppressed SMAD4 (3′UTR targeting). Inhibition of miR-196a-5p reduced invasion and reversed EMT marker profile (↑E-cadherin; ↓N-cadherin/vimentin/slug/snail), while SMAD4 overexpression antagonized EMT/invasion in CD44(+) cells. SMAD4 protein was reduced in GC tissues vs. adjacent and correlated with tumor differentiation/TNM depth parameters.	Predominantly cell-line CSC model (no in vivo metastasis model for miR-196a-5p); clinical dataset centers on SMAD4 IHC, not miR-196a-5p tissue levels; microarray discovery performed in one cell line context (SNU-5 CD44±).
miR-181a [77]	Human + in vitro + in vivo	Human: 90 GC tissues + 30 adjacent non-tumor tissues (adenocarcinoma; no pre-op chemo/RT); survival analysis: high vs. low miR-181a split by median (reported as 45 vs. 45). In vitro: GC cell lines MKN45, SGC-7901, MGC803, BGC-823; normal gastric GES-1; HEK293T luciferase. In vivo: NU/NU nude mice-subcutaneous tumor growth 3 mice/group (1 × 10^6^ cells; 5-week endpoint) and tail-vein lung metastasis 4 mice/group (5 × 10^6^ cells; 12-week endpoint).	qRT-PCR (miR-181a + caprin-1 mRNA); IHC for caprin-1 in tissues; dual-luciferase (caprin-1 3′UTR); WB (caprin-1)	miR-181a normalized to U6; caprin-1 mRNA normalized to β-actin; WB loading control GAPDH	miR-181a was overexpressed in GC tissues and cell lines, associated with larger tumor size, LN/distant metastasis and higher TNM stage; higher miR-181a linked to worse OS. miR-181a downregulation reduced proliferation, migration/invasion and increased apoptosis in vitro, and suppressed xenograft growth and lung metastasis in vivo. caprin-1 confirmed as a direct miR-181a target (luciferase); caprin-1 siRNA rescued the phenotypes.	“Control” tissues are fewer (30) than tumor samples (90) and not explicitly described as paired; mechanistic axis focuses on caprin-1 but EMT-specific marker panel is not a central endpoint; animal models use small group sizes (3-4/group).
miR-616-3p [78]	Human + in vitro	Human: 63 paired GC tumor vs. adjacent non-cancerous tissues (surgery 2008-2009; no pre-op chemo/RT). In vitro: GC cell lines MKN-28, AGS, SGC-7901, MGC-803; normal gastric GES-1; HEK293T for luciferase; HUVECs for tube formation (3 × 10^4^ cells/well; assays ≥ 3 independent experiments).	qRT-PCR (SYBR Premix Ex Taq) on Applied Biosystems Prism 7900; PTEN targeting validated by dual-luciferase reporter assay	miR-616-3p qRT-PCR normalized to U6; WB endogenous control β-actin	miR-616-3p was upregulated in GC tissues and higher expression predicted worse OS and RFS (median cut-off). Gain-/loss-of-function showed miR-616-3p increased migration/invasion and promoted EMT marker changes (E-cadherin↓; vimentin/snail/slug↑). miR-616-3p enhanced angiogenesis (HUVEC tube formation; VEGFA/VEGFR2 ↑). Mechanistically, miR-616-3p directly suppressed PTEN, activating AKT/mTOR; PTEN restoration reversed EMT/angiogenesis effects.	No in vivo tumor/metastasis validation; single-center patient cohort (*n* = 63) with survival stratification by median; mechanistic work mainly relies on cell models and HUVEC tube formation assays.
miR-301a-3p [79]	Human + in vitro + in vivo	Human: primary GC tissues with peritoneal metastasis (*n* = 10) vs. without (*n* = 10); serum exosomes: healthy controls (*n* = 10) vs. GC without peritoneal metastasis (*n* = 10) vs. GC with peritoneal metastasis (*n* = 10). In vitro: GC cell lines (incl. hypoxia-conditioned exosomes), endothelial assays. In vivo: mouse metastasis models (*n* = 5/group).	RT-qPCR (exosomal miRNA + cellular/tissue miRNA quantification)	U6 (cellular/tissue miRNA); cel-miR-39 spike-in	Hypoxic GC cells secrete exosomes enriched in miR-301a-3p, which can be transferred to recipient cells and enhance malignant phenotypes. Mechanistically, miR-301a-3p promotes HIF-1α-driven pro-metastatic signaling by targeting a negative regulator (reported as PHD3), contributing to invasion/metastasis and pro-angiogenic effects. Clinically, serum exosomal miR-301a-3p is higher in peritoneal metastasis compared with non-metastatic GC and controls.	Small clinical cohorts (10 per group). Experimental models use engineered exosome transfer and xenograft/metastasis assays that may not fully reproduce natural metastatic evolution. Limited detail on broader clinical confounders (e.g., treatment effects) within the sampling design.
miR-150 [80]	Human + in vitro + in vivo	Human tissues: paired GC vs. adjacent (*n* = 50 pairs for miR-150); *n* = 34 pairs used for SUFU correlation. Cells: multiple GC cell lines + normal epithelial cells. In vivo: nude mice xenografts (*n* = 6/group), local treatment with miR-150 inhibitor vs. control.	RT-qPCR (miRNA); WB/qPCR for pathway targets; luciferase reporter for target validation	WB loading control GAPDH	miR-150 acts as an oncogenic miRNA in GC: promotes proliferation, migration, and EMT. SUFU is validated as a direct target (3′UTR reporter), and miR-150 activates both Hedgehog and Wnt/β-catenin signaling, consistent with pro-EMT and tumor progression. In vivo, miR-150 inhibition reduced tumor growth (including complete regression in a subset of treated mice).	Survival association not significant in the full TCGA cohort (trend only in late-stage subsets). IHC in vivo was frequently negative due to long-term storage of tumor samples, limiting protein-level validation in tissues. Xenograft work relies on limited models (and mechanistic interpretation is mainly from cell-line experiments).
miR-27a-3p [81]	Human + in vitro	Human miRNA qPCR: 10 paired GC tissues vs. adjacent normal tissues (>3 cm from tumor). Human IHC: 108 GC samples stained for NOVA1 (clinicopath correlations + OS). In vitro: AGS gastric cancer cells (pre-miR-27a-3p overexpression; NOVA1 shRNA knockdown).	RT-qPCR (SYBR Green) using mirVana RT-qPCR miRNA Detection kit; NOVA1 targeting assessed by dual-luciferase reporter assay (NOVA1 3′UTR).	miR-27a-3p qPCR normalized to U6; mRNA qPCR normalized to GAPDH; luciferase normalized by co-transfected Renilla; WB loading control β-actin.	miR-27a-3p was upregulated in GC tissues (10 paired samples) and higher tumor miR-27a-3p was associated with shorter OS (comparison of OS <40 vs. ≥40 months). In AGS cells, miR-27a-3p overexpression induced EMT-like phenotype with E-cadherin/keratin 8 ↓ and N-cadherin/fibronectin ↑. miR-27a-3p directly suppressed NOVA1 protein (3′UTR luciferase + WB) without reducing NOVA1 mRNA, consistent with translational repression. NOVA1 knockdown phenocopied EMT marker changes, and low NOVA1 protein in tumors (IHC, *n* = 108) correlated with lymph node metastasis, TNM stage, and worse OS.	Human miR-27a-3p expression analysis was performed in a small subset (10 pairs) despite a larger IHC cohort for NOVA1; functional validation relies on a single GC cell line (AGS); no in vivo metastasis model and no independent external clinical validation cohort for miR-27a-3p.
miR-192-5p [82]	Human + in vitro + in vivo	Human tissues: 30 paired GC + adjacent normal tissues (miR-192-5p and RB1 qRT-PCR); RB1 WB shown in 6 paired samples. Peripheral blood: 30 GC patients + 40 healthy volunteers. In vitro: GC cell lines BGC-823 + MKN45 (miR-192-5p mimic/inhibitor; RB1 OE/siRNA), PBMC co-culture 1:1 ratio (2 × 10^5^ cells/mL; 96 h). In vivo: (1) BGC-823 xenograft in BALB/c nude mice + intratumoral miR-192-5p antagomir/NC + PBMC implantation; *n* = 5/group. (2) MFC tumor model in C57BL/6 mice with treatment arms incl. antagomir ± anti-CD25 ± anti-IL-10 (flow cytometry of tumor/spleen; *n* = 5/group reported for several readouts).	miRNA quantification by qRT-PCR; target validation by RIP (Ago2) + dual-luciferase (RB1 3′UTR). EMT/axis validation by WB (E-cadherin, vimentin, RB1, p65/p-p65, IL-10), ELISA (secreted IL-10), flow cytometry (CD4+CD25+FOXP3+ Tregs; PD-1+FOXP3+ Tregs), ChIP (p65 binding to miR-192-5p promoter), Co-IP (RB1-p65 interaction).	miR-192-5p qRT-PCR normalized to U6; RB1 mRNA normalized to GAPDH; WB loading control GAPDH. Luciferase assays used reporter constructs (WT/MUT binding sites) with standard internal control design.	miR-192-5p is overexpressed in GC and associated with poor prognosis in their clinical subset. miR-192-5p directly binds RB1 3′UTR and suppresses RB1, promoting EMT (E-cadherin↓, vimentin↑) plus proliferation/migration/invasion. Mechanistically, RB1 restrains NF-κB p65 transcriptional activity; loss of RB1 (via miR-192-5p) increases p65 activity, driving IL-10 secretion. Tumor cells with activated miR-192-5p/RB1 axis increased Treg differentiation and PD-1+ Treg fraction in PBMC co-culture; effects were reduced by IL-10 neutralization or p65 inhibition (BAY11-7082). In vivo, miR-192-5p antagomir reduced tumor growth, decreased IL-10 and Treg infiltration, and shifted EMT markers toward epithelial state.	Single-center human cohort with limited size (30 paired tissues) and heavy reliance on mechanistic cell-line models. Immune conclusions derive from PBMC co-culture and PBMC implantation approaches rather than fully endogenous humanised immunity. Authors note a key limitation: lack of direct corroboration that Treg deficiency inhibits tumor progression by affecting immune cells and tumor cells, and further work is needed to dissect immune mechanisms and PD-1/PD-L1 regulation.
miR-192/miR-215 [29]	Human + in vitro + in vivo	Human (protein-level, IHC): tissue microarray with 90 paired GC + para-cancer tissues; 5-6 years follow-up clinicopath data. “Fresh GC samples” were collected for RNA work, but not specified in the paper. In vitro: BGC-823 (GC) + HFE145 (gastric epithelial) with miR-192/215 mimics/inhibitors; SMG-1 siRNAs. In vivo: subcutaneous BGC-823 xenografts, *n* = 5/group, arms: inhibitor NC vs. miR-192 inhibitor vs. miR-215 inhibitor (local intratumoral injection q3 days × 2 weeks).	No endogenous miRNA profiling platform reported (miR-192/215 were functionally modulated using synthetic mimics/inhibitors, incl. cholesterol-conjugated inhibitors for in vivo). Target discovery used Agilent whole-genome cDNA microarrays (4 × 44 K) in cell models. Target validation: dual-luciferase 3′UTR assay (psiCHECK-2) for SMG-1	Luciferase: firefly normalized to Renilla. WB loading control: β-actin. IHC scored by intensity-based scoring by two blinded pathologists	SMG-1 was identified as a candidate target from microarray screening and confirmed as a direct miR-192/215 target by 3′UTR luciferase and WB (mimics ↓SMG-1; inhibitors ↑SMG-1). In GC tissue microarrays, SMG-1 protein was lower in tumors vs. matched para-cancer tissues, and low SMG-1 was associated with larger tumor size and serosal invasion, though not with OS. Functionally, miR-192/215 inhibition reduced proliferation/colony formation and migration/invasion, while SMG-1 knockdown rescued these inhibitory effects. In vivo, miR-192/215 inhibitors reduced xenograft growth. Mechanistically, SMG-1 loss promoted Wnt pathway activation (↑cyclin D1/CD44/MMP-7) and EMT marker shift (E-cadherin↓, N-cadherin↑), reversible by miR inhibition and SMG-1 siRNA co-transfection.	Human data are protein-level (SMG-1 IHC) without a matched, explicitly reported clinical miR-192/215 quantification in this manuscript. Functional work uses limited models (primarily BGC-823 for loss-of-function and HFE145 for gain-of-function), and the in vivo experiment is a subcutaneous growth model (no orthotopic or metastasis endpoints). Wnt/EMT involvement is inferred from marker changes and downstream targets rather than pathway-wide validation.

↑ indicates increase and ↓ indicates decrease.

## Data Availability

No new data were created or analyzed in this study. Data sharing is not applicable to this article.

## Abbreviations

ABCB1	ATP-binding cassette subfamily B member 1 (P-glycoprotein)
ABL2	Abelson tyrosine-protein kinase 2
AGO	Argonaute protein
AKT	protein kinase B
ALEX1	armadillo repeat-containing protein X-linked 1
AML	acute myeloid leukaemia
AUC	area under the curve
CAF	cancer-associated fibroblast
CAFs	cancer-associated fibroblasts
CCL20	C-C motif chemokine ligand 20
CCL22	C-C motif chemokine ligand 22
CCL5	C-C motif chemokine ligand 5
Cdks	cyclin-dependent kinases
CI	confidence interval
CIN	chromosomal instability
CLL	chronic lymphocytic leukaemia
CRISPR	clustered regularly interspaced short palindromic repeats
CSC	cancer stem cell
Ct	cycle threshold
CXCL8	C-X-C motif chemokine ligand 8 (interleukin-8)
DDP	cisplatin
DFS	disease-free survival
DGCR8	DiGeorge syndrome critical region 8
DICER	endoribonuclease Dicer
DROSHA	Drosha ribonuclease III
ECM	extracellular matrix
EMT	epithelial-mesenchymal transition
EMT-TFs	Epithelial-Mesenchymal Transition Transcription Factors
FAM3C	family with sequence similarity 3 member C
GAS5	growth arrest-specific 5
GC	gastric cancer
GLOBOCAN	Global Cancer Observatory (Global Cancer Statistics database)
GOT1	glutamic-oxaloacetic transaminase 1
GS	genomically stable
*H. pylori*	*Helicobacter pylori*
HGF	hepatocyte growth factor
HIF-1α	hypoxia-inducible factor 1-alpha
IFN-γ	interferon gamma
IGF	insulin-like growth factor
IL-17A	interleukin-17A
IL-1β	interleukin-1 beta
IL-6	interleukin-6
IL-8	interleukin-8
ILEI	interleukin-like EMT inducer
JAK/STAT	Janus kinase/signal transducer and activator of transcription pathway
JAK2/STAT3	Janus kinase 2/signal transducer and activator of transcription
JNK	c-Jun N-terminal kinase
lncRNA	long non-coding RNA
MALT	mucosa-associated lymphoid tissue
MAPK	mitogen-activated protein kinase
MAPK1	mitogen-activated protein kinase 1
MET	mesenchymal-epithelial transition
MIF	macrophage migration inhibitory factor
miRNA	microRNA
MMPs	matrix metalloproteinases
mRNA	messenger RNA
MSI	microsatellite instability
mTOR	mechanistic target of rapamycin
ncRNA	non-coding RNA
NET	neutrophil extracellular trap
NK	natural killer (cell)
NKG2D	natural killer group 2D receptor
NLR	neutrophil-to-lymphocyte ratio
NTN4	netrin-4
PD-1	programmed cell death protein 1
PD-L1	programmed death-ligand 1
PI3K	phosphatidylinositol 3-kinase
PLR	platelet-to-lymphocyte ratio
pre-miRNA	precursor microRNA
pri-miRNA	primary microRNA transcript
PTEN	phosphatase and tensin homolog
qRT-PCR	quantitative reverse transcription PCR
RISC	RNA-induced silencing complex
sens	sensitivity
SNAI2	Snail family transcriptional repressor 2 (Slug)
spec	specificity
TAM	tumor-associated macrophage
TAN	tumor-associated neutrophil
TCGA	The Cancer Genome Atlas
TGF-β	transforming growth factor beta
TNF	tumor necrosis factor
TNF-α	tumor necrosis factor alpha
TNM	tumor-node-metastasis (staging system)
Treg	regulatory T cell
UTR	untranslated region
VEGF	vascular endothelial growth factor
VEGF-C	vascular endothelial growth factor C
WGS	whole-genome sequencing
WT	wild-type
